# Huoxue Huatan Decoction Ameliorates Myocardial Ischemia/Reperfusion Injury in Hyperlipidemic Rats *via* PGC-1α–PPARα and PGC-1α–NRF1–mtTFA Pathways

**DOI:** 10.3389/fphar.2020.546825

**Published:** 2020-09-15

**Authors:** Fei Lin, Yu-Qing Tan, Xuan-Hui He, Li-Li Guo, Ben-Jun Wei, Jun-Ping Li, Zhong Chen, Heng-Wen Chen, Jie Wang

**Affiliations:** ^1^ Heart Center of Xinxiang Medical University, The First Affiliated Hospital of Xinxiang Medical University, Xinxiang, China; ^2^ Department of Cardiology, Guang’anmen Hospital, China Academy of Chinese Medical Sciences, Beijing, China; ^3^ Graduate School, Beijing University of Chinese Medicine, Beijing, China; ^4^ Key Laboratory of Ministry of Education Department of Lanzhou Province and Dunhuang Medical Transformation, Gansu University of Chinese Medicine, Lanzhou, China

**Keywords:** Huoxue Huatan Decoction, hyperlipidemia, ischemia/reperfusion (I/R), mitochondria, PGC-1α

## Abstract

**Objective:**

The aim of this study was to eluc\idate the preventive and therapeutic effects and the underlying mechanisms of Huoxue Huatan Decoction (HXHT) on myocardial ischemia/reperfusion (I/R) injury in hyperlipidemic rats.

**Methods:**

An I/R model was established in hyperlipidemic Wistar rats. After 4–8 weeks of HXHT treatment, the physical signs of rats were observed. Lipid metabolism, myocardial enzyme spectrum, cardiac function, myocardial histomorphology, and mitochondrial biosynthesis were investigated by a biochemical method, ultrasonography, electron microscopy, pathological examination, real-time PCR, and Western blot.

**Results:**

HXHT can affect lipid metabolism at different time points and significantly reduce the levels of cholesterol (CHO), triglyceride (TG), high-density lipid-cholesterol (HDL-C), and low-density lipid-cholesterol (LDL-C) in hyperlipidemic rats (*P* < 0.05 or *P* < 0.01); it can significantly reduce the levels of creatine kinase-MB (CK-MB) and lactate dehydrogenase (LDH), reduce the myocardial infarct size and myocardial ischemic area, and improve cardiac function. The results of myocardial histomorphology showed that HXHT could protect myocardial cells, relieve swelling, reduce the number of cardiac lipid droplets, and improve myocardial mitochondrial function. HXHT could significantly increase the levels of total superoxide dismutase (T-SOD) and succinate dehydrogenase (SDH) (*P* < 0.05 or *P* < 0.01), increase CuZn-superoxide dismutase (CuZn-SOD) and glutathione-peroxidase (GSH-Px) levels, and decrease the levels of malondialdehyde (MDA) (*P* < 0.05); it could increase the mRNA and protein expression levels of peroxisome proliferator-activated receptor-gamma coactivator 1 alpha (PGC-1α), peroxisome proliferator-activated receptor alpha (PPARα), nuclear respiratory factor 1 (NRF1), and mitochondrial transcription factor A (mtTFA) (*P* < 0.05 or *P* < 0.01), and increase the synthesis of mitochondrial DNA (mtDNA) (*P* < 0.01).

**Conclusion:**

HXHT can reduce myocardial I/R injury in hyperlipidemic rats. The protective mechanisms may involve a reduction in blood lipids, enhancement of PGC-1α–PPARα pathway activity, and, subsequently, an increase in fatty acid β-oxidation, which may provide the required input for mitochondrial energy metabolism. HXHT can additionally enhance PGC-1α–NRF1–mtTFA pathway activity and, subsequently, increase the antioxidant capacity, promote mtDNA synthesis, and reduce mitochondrial damage. The two pathways use PGC-1α as the intersection point to protect mitochondrial structure and function, reduce I/R-induced injury, and improve cardiac function.

Coronary heart disease refers to heart disease caused by hypoxia and ischemia of the myocardium due to arterial obstruction or stenosis, which is a common type of disease caused by coronary atherosclerosis (Benjamin et al., 2018). Hyperlipidemia is closely related to the risk of coronary heart disease. At present, 50.0% of patients in China have hypertension, of whom 37.5% have coronary heart disease and more than 30.0% have peripheral arterial disease (Chen et al., 2014). The risk factors of cardiovascular disease can be broadly divided into two categories, i.e., intervenable and non-intervenable. Intervenable factors include lifestyle (smoking, blood pressure, blood sugar, and cholesterol) and medication, non-intervenable factors include age, gender, race, and family history (Derosa et al., 2020).

A high-fat diet, as a common intervenable factor, can lead to lipid accumulation, induce the inflammatory response, promote atherosclerosis, and accelerate the development of cardiovascular diseases (Cao et al., 2018). Specifically, a high-fat diet can increase plasma low-density lipoprotein (LDL) levels. LDL is oxidized and modified into oxidized LDL (Ox-LDL). Monocytes take up Ox-LDL, and the expression of adhesion molecules increases. A high-fat diet promotes the conversion of monocytes into foam cells, which is a key step in the development of atherosclerosis (Henning et al., 2018). Chronic arterial inflammation plays an important role in the pathogenesis of atherosclerosis. The secretion of atherogenic cytokines induces the expression of endothelial adhesion molecules, which mediate the attachment of monocytes and lymphocytes (Ham et al., 2019). Atherosclerosis results in thickening of the vessel wall and a reduction of lumen diameter and blood flow. The current guidelines mainly focus on the treatment of hyperlipidemia, with LDL and cholesterol levels as the main indicators (Okopień et al., 2016), and the ultimate goal is to reduce the risk of atherosclerotic cardiovascular disease. The main ways of lowering lipids are lifestyle regulation and pharmacological lipid-lowering. Studies have shown that reducing LDL-cholesterol (LDL-C) and triglyceride (TG) levels, reducing the intake of saturated fatty acids and refined carbohydrates, and increasing the intake of fruits, vegetables, cereals, and low-fat dairy products can reduce the risk of cardiovascular events and prevent coronary plaque progression. It is important to focus on whole foods and eating habits to successfully reduce the risk of cardiovascular disease (Alissa and Ferns, 2017; Welty, 2020). Pharmacological treatment is still mainly based on statins and targeted at LDL-C (Han et al., 2019).

Traditional Chinese medicine has a rich clinical history of coronary heart disease and dyslipidemia treatment. Coronary heart disease accompanied by elevated blood lipids or obesity is classified as coronary heart disease with phlegm–blood stasis syndrome, and both TG and LDL-C levels are significantly increased in patients with coronary heart disease with phlegm–blood stasis syndrome (Wang et al., 2008). The only patented medicines for the treatment of coronary heart disease with phlegm–blood stasis syndrome in China are Danlou tablets (The cardiovascular disease branch of China Association of Chinese Medicine, 2019). Mitochondria, as semi-autonomous organelles, are known as “enzyme bags” and “power plants.” They are the main sites for the biological oxidation of sugars, proteins, and fats. Myocardial cells have a high demand for energy, and mitochondria account for approximately 40% (m/m) of cardiomyocytes. Abnormal structure and function of mitochondria directly affect myocardial energy metabolism, oxidative stress, ion uptake and release, and apoptosis (Goldenthal, 2016).

Huoxue Huatan Decoction (HXHT) is a commonly prescribed medicine for the treatment of coronary heart disease with phlegm–blood stasis syndrome, and has been used clinically for more than 20 years. It is composed of *Salvia miltiorrhiza* Bunge, *Astragalus mongholicus* Bunge, *Panax notoginseng* (Burkill) F.H. Chen, *Ginkgo biloba* L., *Trichosanthes kirilowii* Maxim., *Allium macrostemon* Bunge, and *Ziziphus jujuba* Mill. HXHT has the effects of invigorating qi and activating blood, resolving phlegm, and removing blood stasis.

The main components of *S. miltiorrhiza* Bunge are tanshinone IIA and *S.* miltiorrhiza polyphenols, which have antioxidant, anti-inflammatory, anticoagulant, anti-atherosclerosis, and vasodilator effects. They can also protect the myocardium and vascular endothelium, reduce adipogenesis, and reduce the proliferation and migration of vascular smooth muscle cells (Ren et al., 2019; Yuan et al., 2020). Astragaloside IV is the main active ingredient of *A. mongholicus* Bunge. It has protective effects on ischemic injury and cardiovascular disease. It has anti-inflammatory, immunomodulatory, and antioxidant effects, and exerts cardioprotective effects through multiple signaling pathways (Li et al., 2017). The main active ingredient of *P. notoginseng* (Burkill) F.H. Chen is *P. notoginseng* saponins, and its pharmaceutical preparations, such as Xueshuantong, Xuesaitong, and Naodesheng, are widely used and have cardiovascular protective effects. *P. notoginseng* saponins can reduce the formation of atherosclerotic lesions (Liu et al., 2019; Liu et al., 2020). *G. biloba* L. can effectively improve metabolic syndrome, diabetes, hypertension, and dyslipidemia, reduce the risk of cardiovascular disease (Eisvand et al., 2020), and alleviate vascular aging-related dysfunction (Li et al., 2020). *T. kirilowii* Maxim. and *A. macrostemon* Bunge often appear as a pair in commonly used drugs, which are essential for the treatment of angina pectoris, heart failure, and myocardial infarction. *T. kirilowii* Maxim. can protect the myocardium and endothelial cells, eliminates phlegm, and has anti-oxidation, anticoagulation, and anti-inflammatory effects (Yu et al., 2018). *A. macrostemon* Bunge is a potential drug to treat hyperglycemia, hyperlipidemia, and visceral obesity (Xie et al., 2008), and can also reduce myocardial ischemic injury by regulating abnormal energy metabolism (Li et al., 2014). *Z. jujuba* Mill. has sedative and hypnotic effects, and it is clinically used to treat insomnia, forgetfulness, and dizziness. In addition, it also has anti-inflammatory, anti-oxidation, blood pressure-lowering, and blood lipid-lowering effects (He et al., 2020), and it can protect myocardial cells from acute cardiac ischemia/reperfusion (I/R) injury (Gu et al., 2019). The compatibility of the whole prescription is reasonable, it has immune regulatory effects, and it can protect myocardial cells from ischemia and hypoxia. Previous clinical and experimental studies have shown that this prescription can significantly improve the clinical symptoms of patients with coronary heart disease with phlegm–blood stasis syndrome, with good safety (Li, 2016; Zhang et al., 2016).

Therefore, in this study, we analyze the effects of HXHT on myocardial I/R injury in hyperlipidemic rats from the perspectives of lipid metabolism, the myocardial enzyme spectrum, myocardial histomorphology, and myocardial infarct size. In addition, the underlying mechanisms of HXHT in preventing and treating myocardial I/R in hyperlipidemic rats are studied from the perspectives of myocardial mitochondrial function and the expression of genes related to mitochondrial biogenesis.

## Materials and Methods

### Preparation of Experimental Drugs

HXHT was provided by the Pharmacology Laboratory of Traditional Chinese Medicine, Guang’anmen Hospital, China Academy of Chinese Medical Science. It is composed of *S. miltiorrhiza* Bunge (17.4%, Specimen ID 33873, product batch number 140381391), *A. mongholicus* Bunge (7.0%, Specimen ID 106823, product batch number 140581231), *P. notoginseng* (Burkill) F.H. Chen (17.4%, Specimen ID 69689, product batch number 140581091), *G. biloba* L. (11.6%, Specimen ID 4988, product batch number 140581421), *T. kirilowii* Maxim. (17.4%, Specimen ID 15439, product batch number 140281611), *A. macrostemon* Bunge (17.4%, Specimen ID 50154, product batch number 140580301), and *Z. jujuba* Mill. (11.6%, Specimen ID 27570, product batch number 140581941) (**Table 1**). All of the medicinal names have been unified using the Kew Medicinal Plant Names Service. All of the specimens have been deposited in the Chinese National Herbarium, Institute of Botany, Chinese Academy of Sciences (20 Nanxincun, Xiangshan, Beijing 100093, China). All of the medicinal materials were provided by Beijing Kangmei Pharmaceutical Co., Ltd. (Beijing, China).

**Table 1:** The composition of traditional Chinese medicine in Huoxue Huatan Decoction (HXHT).

Component	Component (Chinese)	Medicinal parts	Percentage (%)	Active ingredient content (%)
*Salvia miltiorrhiza* Bunge	Danshen	Dry roots and rhizomes	17.4	tanshinone IIA 0.31%, cryptotanshinone 0.25%, salvianolic acid B 3.44%
*Astragalus mongholicus* Bunge	Huangqi	Dry roots	7.0	astragaloside IV 0.06%, calycosin glucoside 0.04%
*Panax notoginseng* (Burkill) F.H. Chen	Sanqi	Dry roots and rhizomes	17.4	ginsenoside Rg1 5.26%, ginsenoside Rb1 4.00%, notoginsenoside R1 1.09%
*Ginkgo biloba* L.	Yinxingye	Dry leaves	11.6	total flavonols 0.41%, total lactones 0.27%
*Trichosanthes kirilowii* Maxim.	Gualou	Dry ripe fruit	17.4	–
*Allium macrostemon* Bunge	Xiebai	Dry bulb	17.4	–
*Ziziphus jujuba* Mill.	Suanzaoren	Dry mature seeds	11.6	jujuboside A 0.08% spinosin 0.13%

*S. miltiorrhiza* Bunge was extracted 10 times with 60% ethanol through heating reflux twice, each time for 1.5 h. The ethanol was recovered from the extract and concentrated to thick paste (50°C, density 1.20 g/cm³) under reduced pressure, and the concentrated solution was separated twice using an AB-8 macroporous adsorption resin column (Tianjin NANDA Resin Technology Co., Ltd.), where 50% ethanol of the same volume as the column bed was used for elution. The eluate was concentrated into a thick paste (50°C, density 1.20 g/cm³), and dried in vacuum at 60°C to obtain the *S. miltiorrhiza* Bunge extract. The leaves of *Ginkgo biloba* L. were extracted 8 times with 60% ethanol through heating reflux twice, each time for 2 h. After vacuum concentration, the concentrated solution was separated three times using an AB-8 macroporous adsorption resin column, where 70% ethanol of the same volume as the column bed was used for elution. The eluate was concentrated into a thick paste (50°C, density 1.20 g/cm³), and dried in vacuum at 60°C to obtain the *Ginkgo biloba* L. extract. Then 10 volumes of water were added to the remaining material, and the medicine was extracted twice, concentrated (50°C, density 1.20 g/cm³), and dried in vacuum at 60°C. Finally, all the extracts were crushed and mixed to obtain the HXHT extract.

**Figure 1:**
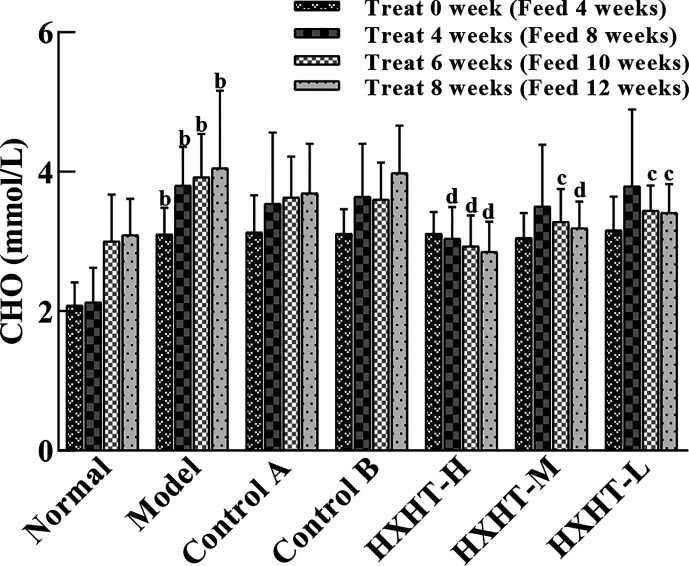
Effects of Huoxue Huatan Decoction (HXHT) on cholesterol (CHO) serum levels in hyperlipidemic rats $\left(\bar{x}\pm s\right)$. The num ber of animals per group at 4 weeks is *n* = 24; the number of animals per group at 8 weeks is *n* = 16. ^b^ *P* < 0.01 compared with the Normal group; ^c^ *P* < 0.05, ^d^ *P* < 0.01 compared with the Model group. HXHT, Huoxue Huatan Decoction; CHO, cholesterol.

**Figure 2:**
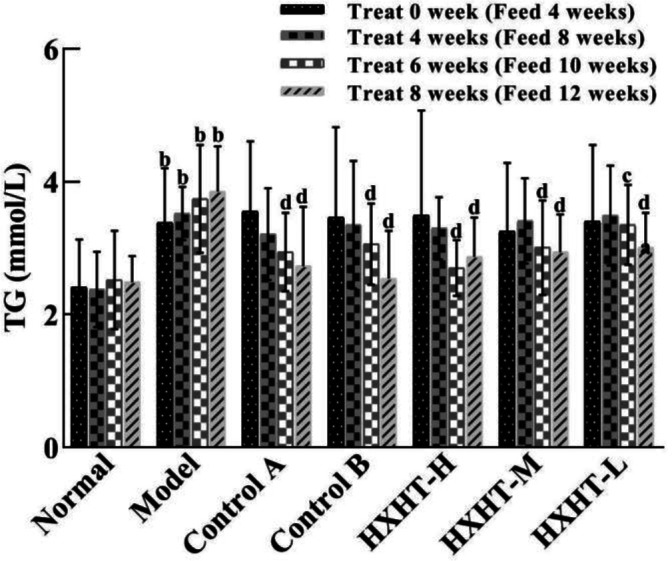
Effects of Huoxue Huatan Decoction (HXHT) on triglyceride (TG) serum levels in hyperlipidemic rats $\left(\bar{x}\pm s\right)$. The number of animals per group at 4 weeks is *n* = 24; the number of animals per group at 8 weeks is *n* = 16. ^b^ *P* < 0.01 compared with the Normal group; ^c^ *P* < 0.05, ^d^ *P* < 0.01 compared with the Model group. HXHT, Huoxue Huatan Decoction; TG, triglyceride.

**Figure 3:**
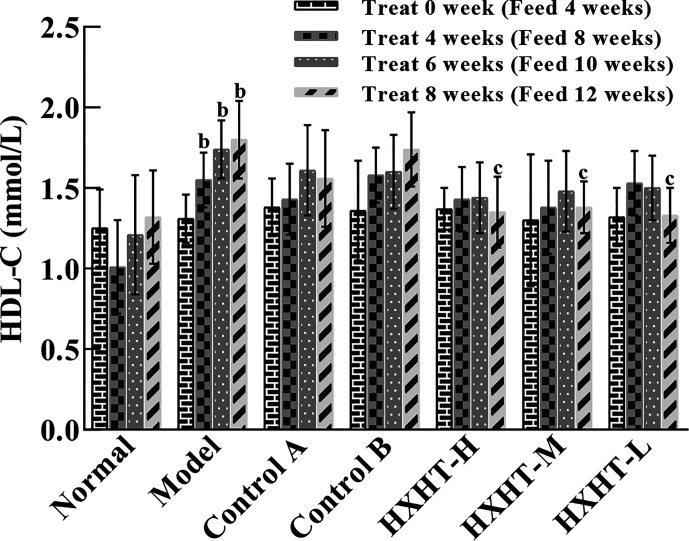
Effects of Huoxue Huatan Decoction (HXHT) on high-density lipid-cholesterol (HDL-C) serum levels in hyperlipidemic rats $\left(\bar{x}\pm s\right)$. The number of animals per group at 4 weeks is *n* = 24; the number of animals per group at 8 weeks is *n* = 16. ^b^ *P* < 0.01 compared with the Normal group; ^c^ *P* < 0.05 compared with the Model group. HXHT, Huoxue Huatan Decoction; HDL-C, high-density lipid-cholesterol.

**Figure 4:**
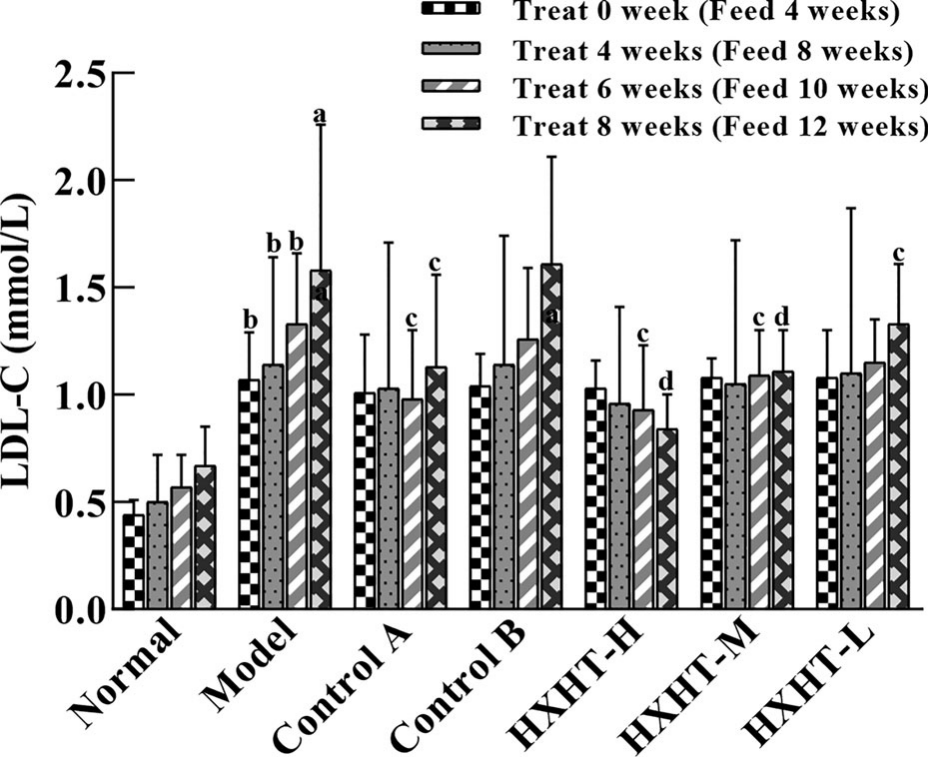
Effects of Huoxue Huatan Decoction (HXHT) on low-density lipid-cholesterol (LDL-C) serum levels in hyperlipidemic rats $\left(\bar{x}\pm s\right)$. The number of animals per group at 4 weeks is *n* = 24; the number of animals per group at 8 weeks is *n* = 16. ^a^ *P* < 0.05, ^b^ *P* < 0.01 compared with the Normal group; ^c^ *P* < 0.05, ^d^ *P* < 0.01 compared with the Model group. HXHT, Huoxue Huatan Decoction; LDL-C, low-density lipid-cholesterol.

**Figure 5:**
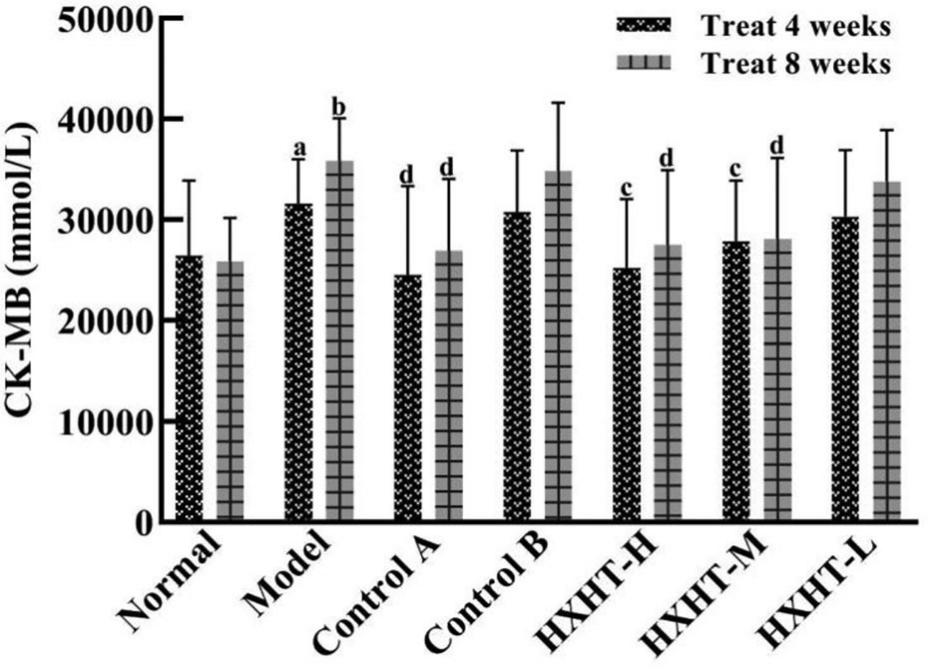
Effects of Huoxue Huatan Decoction (HXHT) on creatine kinase-MB (CK-MB) serum levels in hyperlipidemic ischemia/reperfusion (I/R) rats $\left(\bar{x}\pm s\right)$ The number of animals per group at 4 weeks is *n* = 24; the number of animals per group at 8 weeks is *n* = 16. ^a^ *P* < 0.05, ^b^ *P* < 0.01 compared with the Normal group; ^c^ *P* < 0.05, ^d^ *P* < 0.01 compared with the Model group.

**Figure 6:**
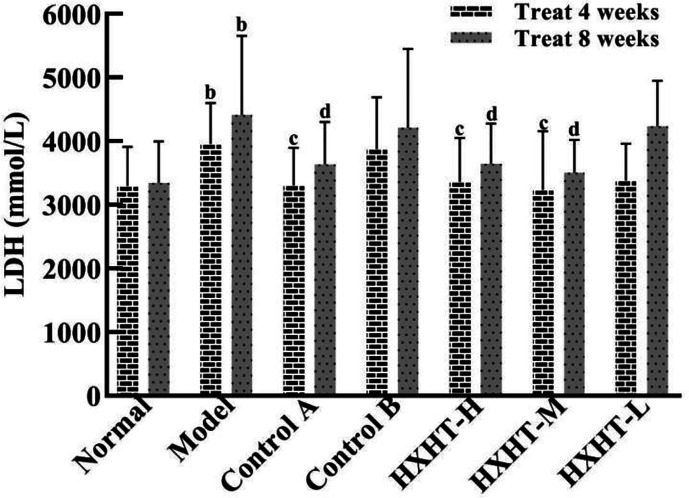
Effects of Huoxue Huatan Decoction (HXHT) on lactate dehydrogenase (LDH) serum levels in hyperlipidemic ischemia/reperfusion (I/R) rats $\left(\bar{x}\pm s\right)$. The number of animals at 4 weeks is *n*_Normal_ = 8; *n*_Model_ = 7; *n*_Control A_ = 7; *n*_Control B_ = 8; *n*_HXHT-H_ = 7; *n*_HXHT-M_ = 8; *n*_HXHT-L_ = 8. The number of animals at 8 weeks is *n*_Normal_ = 16; *n*_Model_ = 15; *n*_Control A_ = 16; *n*_Control B_ = 15; *n*_HXHT-H_ = 16; *n*_HXHT-M_ = 16; *n*_HXHT-L_ = 15. ^b^ *P* < 0.01 compared with the Normal group; ^c^ *P* < 0.05, ^d^ *P* < 0.01 compared with the Model group. HXHT, Huoxue Huatan Decoction; CK-MB, creatine kinase-MB; LDH, lactate dehydrogenase; I/R, ischemia/reperfusion.

**Figure 7:**
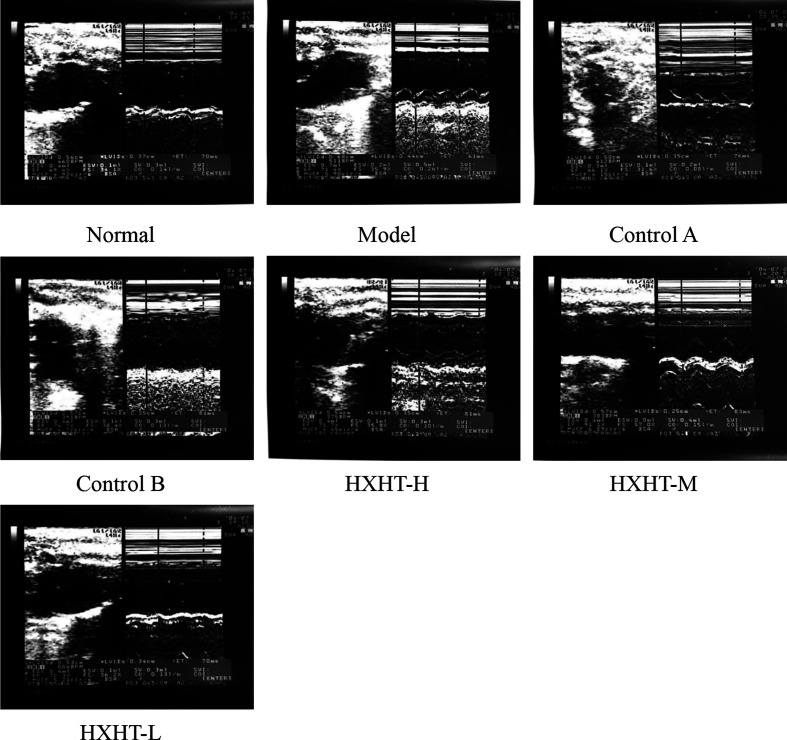
B-ultrasound of hyperlipidemic ischemia/reperfusion (I/R) rats.

**Figure 8:**
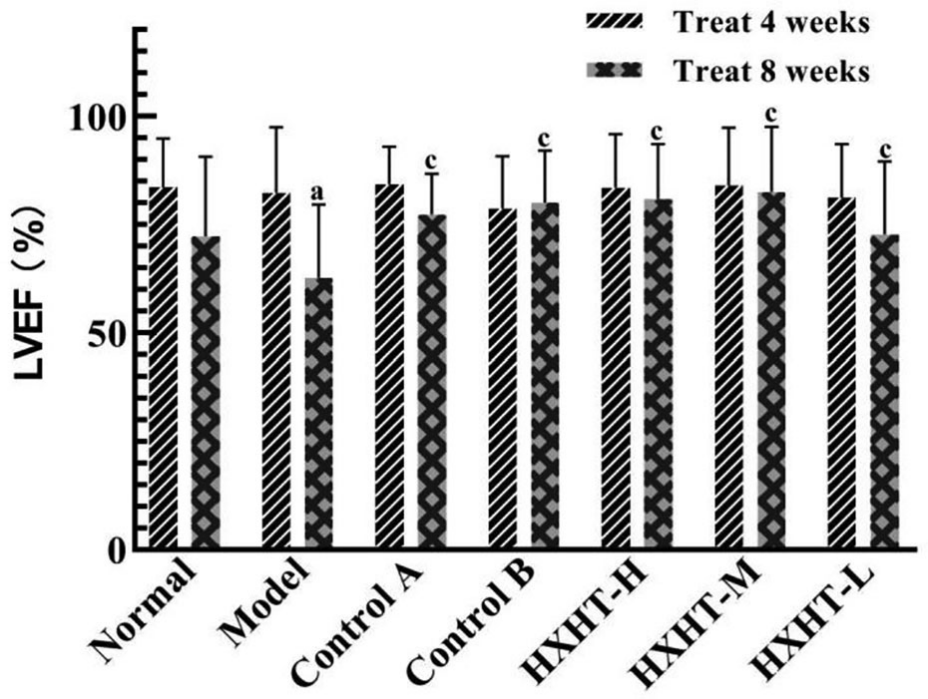
Effects of Huoxue Huatan Decoction (HXHT) on left ventricular ejection fraction (LVEF) in hyperlipidemic ischemia/reperfusion (I/R) rats $\left(\bar{x}\pm s\right)$. TThe number of animals at 4 weeks is *n*_Normal_ = 8; *n*_Model_ = 7; *n*_Control_ A = 7; *N*_Control_ B = 8; *n*_HXHT-H_ = 7; *n*_HXHT-M_ = 8; *n*_HXHT-L_ = 8. The number of animals at 8 weeks is *n*_Normal_ = 16; *n*_Model_ = 15; *n*_Control_ A = 16; *n*_Control_ B = 15; *n*_HXHT-H_ = 16; *n*_HXHT-M_ = 16; *n*_HXHT-L_ = 15. ^a^ *P* < 0.05 compared with the Normal group; ^c^ *P* < 0.05 compared with the Model group.

**Figure 9:**
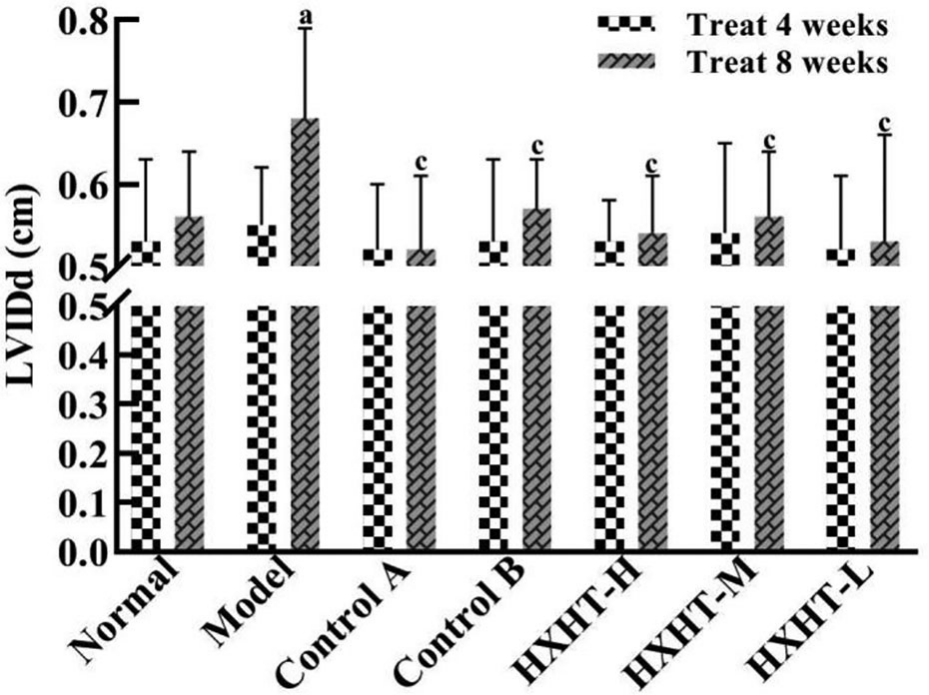
Effects of Huoxue Huatan Decoction (HXHT) on left ventricular end-diastolic inner diameter (LVIDd) in hyperlipidemic ischemia/reperfusion (I/R) rats $\left(\bar{x}\pm s\right)$. The number of animals at 4 weeks is *n*_Normal_ = 8; *n*_Model_ = 7; *n*_Control_ A = 7; *N*_Control_ B = 8; *n*_HXHT-H_ = 7; *n*_HXHT-M_ = 8; *n*_HXHT-L_ = 8. The number of animals at 8 weeks is *n*_Normal_ = 16; *n*_Model_ = 15; *n*_Control_ A = 16; *n*_Control_ B = 15; *n*_HXHT-H_ = 16; *n*_HXHT-M_ = 16; *n*_HXHT-L_ = 15. ^a^ *P* < 0.05 compared with the Normal group; ^c^ *P* < 0.05 compared with the Model group.

**Figure 10:**
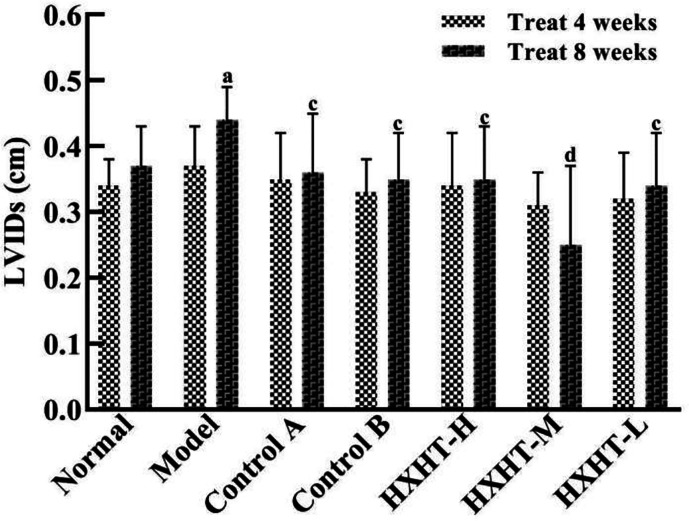
Effects of Huoxue Huatan Decoction (HXHT) on left ventricular end-systolic inner diameters (LVIDs) in hyperlipidemic ischemia/reperfusion (I/R) rats $\left(\bar{x}\pm s\right)$. The number of animals at 4 weeks is *n*_Normal_ = 8; *n*_Model_ = 7; *n*_Control A_ = 7; *n*_Control B_ = 8; *n*_HXHT-H_ = 7; *n*_HXHT-M_ = 8; *n*_HXHT-L_ = 8. The number of animals at 8 weeks is *n*_Normal_ = 16; *n*_Model_ = 15; *n*_Control A_ = 16; *n*_Control B_ = 15; *n*_HXHT-H_ = 16; *n*_HXHT-M_ = 16; *n*_HXHT-L_ = 15. ^a^ *P* < 0.05 compared with the Normal group; ^c^ *P* < 0.05, ^d^ *P* < 0.01 compared with the Model group.

**Figure 11:**
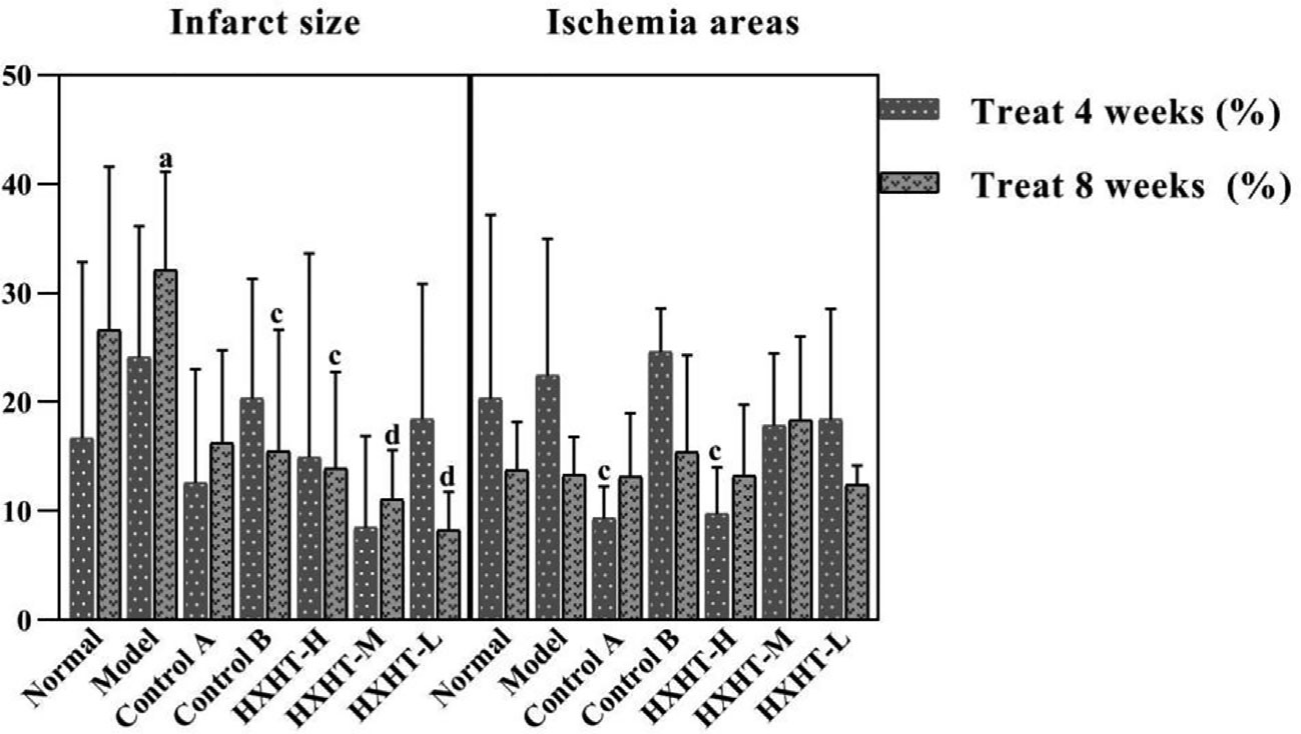
Effects of Huoxue Huatan Decoction (HXHT) on myocardial infarct size and ischemic area in hyperlipidemic ischemia/reperfusion (I/R) rats $\left(\bar{x}\pm s\right)$. The number of animals at 4 weeks is *n*_Normal_ = 8; *n*_Model_ = 7; *n*_Control A_ = 7; *n*_Control B_ = 8; *n*_HXHT-H_ = 7; *n*_HXHT-M_ = 8; *n*_HXHT-L_ = 8. The number of animals at 8 weeks is *n*_Normal_ = 8; *n*_Model_ = 8; *n*_Control A_ = 8; *n*_Control B_ = 7; *n*_HXHT-H_ = 8; *n*_HXHT-M_ = 8; *n*_HXHT-L_ = 7. ^a^ *P* < 0.05 compared with the Normal group; ^c^ *P* < 0.05, ^d^ *P* < 0.01 compared with the Model group. HXHT, Huoxue Huatan Decoction; I/R, ischemia/reperfusion.

**Figure 12:**
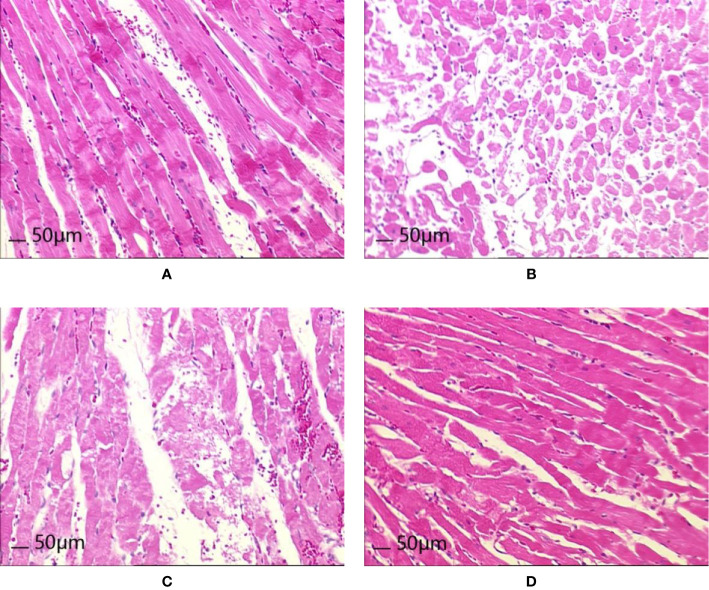
Effects of Huoxue Huatan Decoction (HXHT) on heart histopathology after myocardial ischemia/reperfusion (I/R) injury in hyperlipidemic rats (HE staining, 200×). **(A)** Normal control; **(B)** Model control; **(C)** Control A; **(D)** HXHT-M. The number of animals at 8 weeks is *n*_Normal_ = 8; *n*_Model_ = 7; *n*_Control A_ = 8; *n*_HXHT-M_ = 8. HXHT, Huoxue Huatan Decoction; I/R, ischemia/reperfusion.

**Figure 13:**
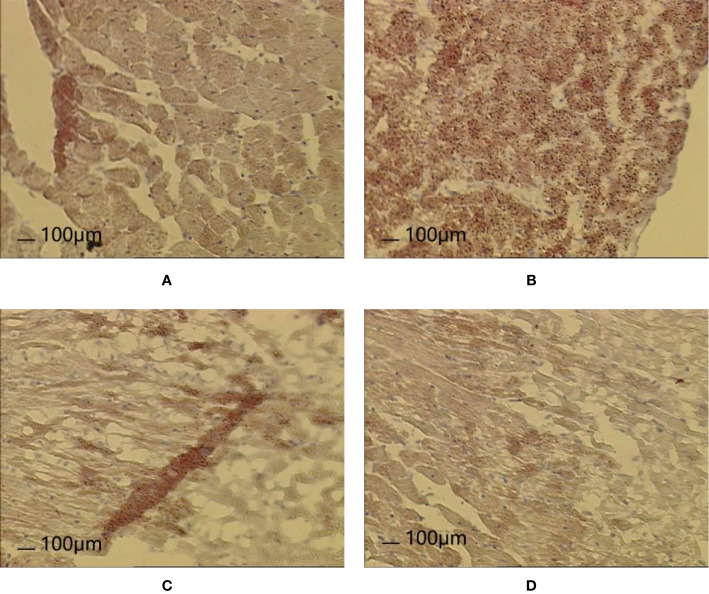
Effects of Huoxue Huatan Decoction (HXHT) on heart histopathology after myocardial ischemia/reperfusion (I/R) injury in hyperlipidemic rats (Oil Red O staining, 100×). **(A)** Normal control; **(B)** Model control; **(C)** Control A; **(D)** HXHT-M. The number of animals at 8 weeks is *n*_Normal_ = 8; *n*_Model_ = 7; *n*_Control A_ = 8; *n*_HXHT-M_ = 8. HXHT, Huoxue Huatan Decoction; I/R, ischemia/reperfusion.

**Figure 14:**
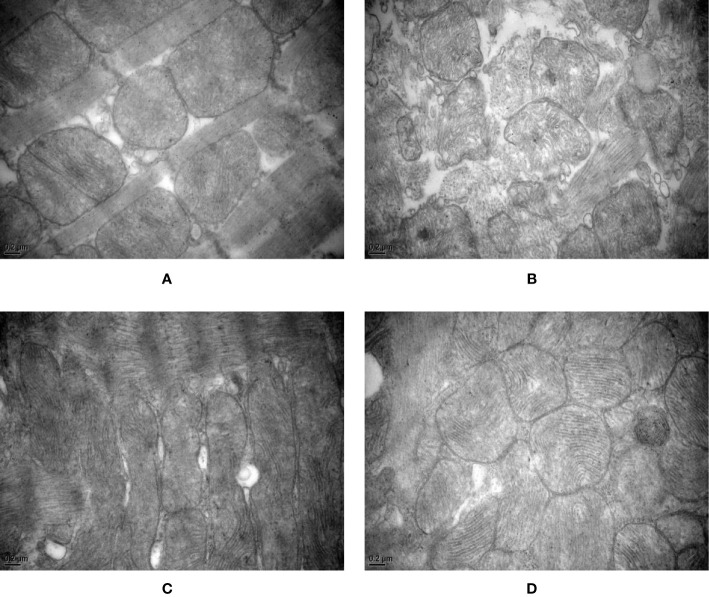
Effects of Huoxue Huatan Decoction (HXHT) on myocardial mitochondria after myocardial ischemia/reperfusion (I/R) injury in hyperlipidemic rats (transmission electron microscopy, 40,000×). **(A)** Normal control; **(B)** Model control; **(C)** Control A; **(D)** HXHT-M. The number of animals at 8 weeks is *n*_Normal_ = 8; *n*_Model_ = 7; *n*_Control A_ = 8; *n*_HXHT-M_ = 8. HXHT, Huoxue Huatan Decoction; I/R, ischemia/reperfusion.

**Figure 15:**
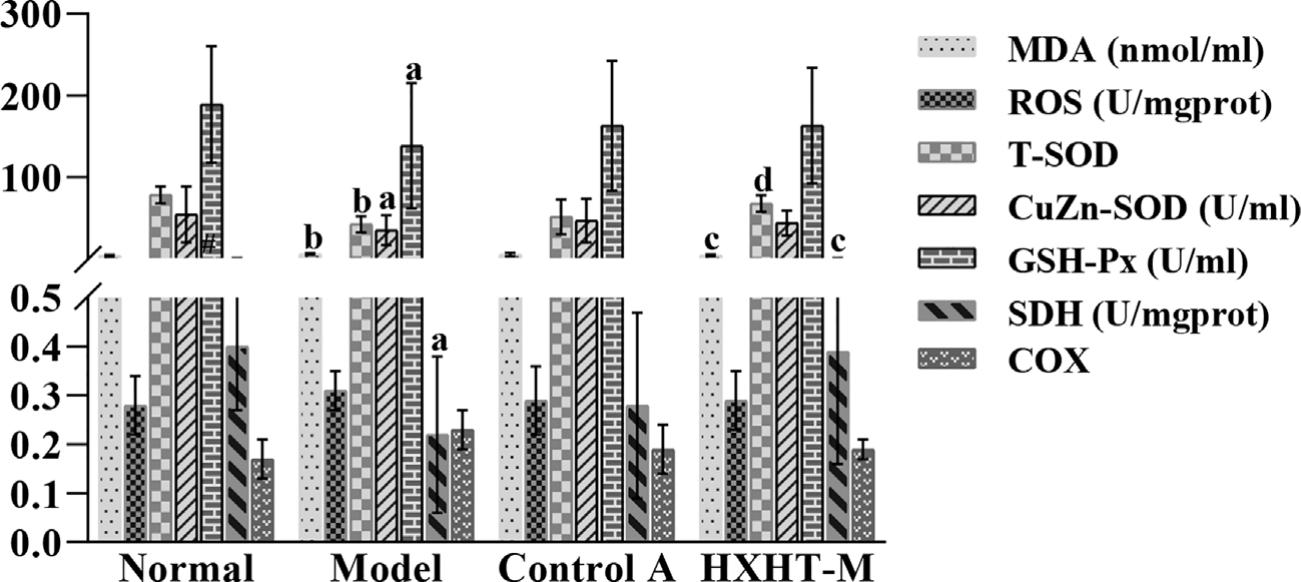
Effects of Huoxue Huatan Decoction (HXHT) on oxidative stress indicators in hyperlipidemic ischemia/reperfusion (I/R) rats. ^a^ *P* < 0.05, ^b^ *P* < 0.01 compared with the Normal group; ^c^ *P* < 0.05, ^d^ *P* < 0.01 compared with the Model group. The number of animals at 8 weeks is *n*_Normal_ = 8; *n*_Model_ = 7; *n*_Control A_ = 8; *n*_HXHT-M_ = 8. HXHT, Huoxue Huatan Decoction; I/R, ischemia/reperfusion.

**Figure 16:**
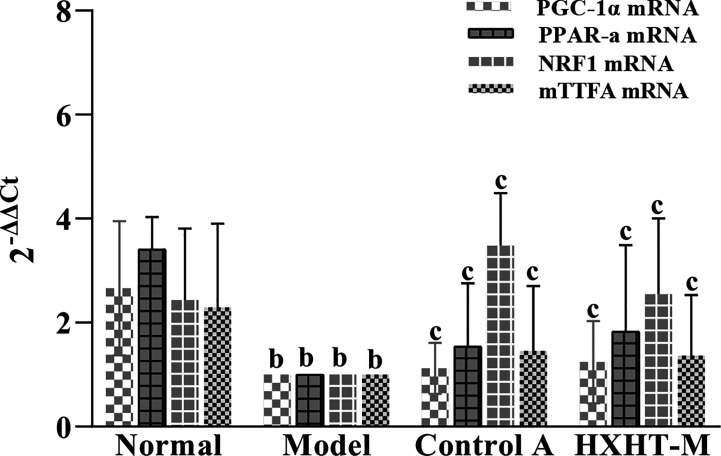
Effects of Huoxue Huatan Decoction (HXHT) on mRNA levels in hyperlipidemic ischemia/reperfusion (I/R) rats $\left(\bar{x}\pm s\right)$. The results were calculated using the 2^−ΔΔCt^ method, where 2^−ΔΔCt^ = (Ct_target gene_ − Ct_housekeeper gene_) experimental group − (Ct_target gene_ − Ct_housekeeper gene_) control group. The amplification factors are presented for comparison reasons. ^b^ *P* < 0.01 compared with the Normal group; ^c^ *P* < 0.05 compared with the Model group. The number of animals at 8 weeks is *n*_Normal_ = 8; *n*_Model_ = 7; *n*_Control A_ = 8; *n*_HXHT-M_ = 8. HXHT, Huoxue Huatan Decoction; I/R, ischemia/reperfusion.

**Figure 17:**
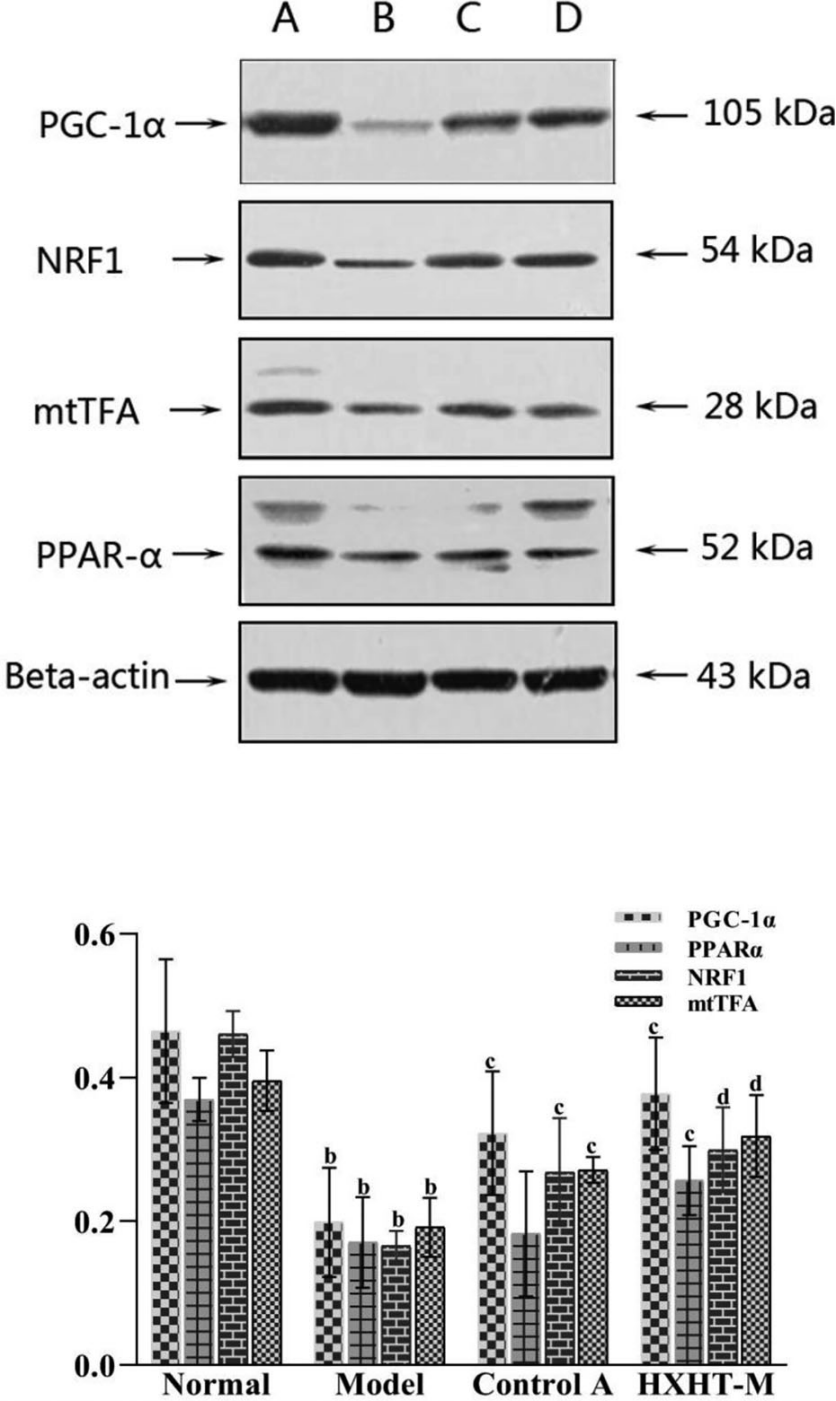
Effects of Huoxue Huatan Decoction (HXHT) on proteins in hyperlipidemic ischemia/reperfusion (I/R) rats $\left(\bar{x}\pm s\right)$. The images were scanned and analyzed with ImageJ software. The gray-scale value was calculated for each band. The relative content of each target protein was calculated by dividing the gray-scale value of the target protein by the gray-scale value of β-actin. ^b^ *P* < 0.01 compared with the Normal group; ^c^ *P* < 0.05, ^d^ *P* < 0.01 compared with the Model group. The number of animals at 8 weeks is *n*_Normal_ = 8; *n*_Model_ = 7; *n*_Control A_ = 8; *n*_HXHT-M_ = 8. HXHT. **(A)** Normal control; **(B)** Model control; **(C)** Control A; **(D)** HXHT-M. HXHT, Huoxue Huatan Decoction; I/R, ischemia/reperfusion.

**Figure 18:**
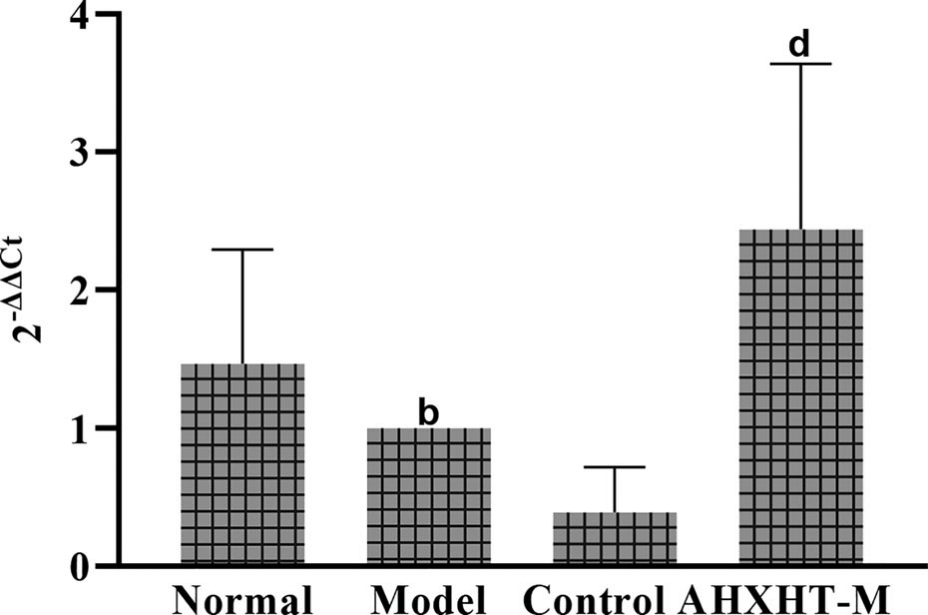
Effects of Huoxue Huatan Decoction (HXHT) on mitochondrial DNA (mtDNA) copy number in hyperlipidemic ischemia/reperfusion (I/R) rats $\left(\bar{x}\pm s\right)$. ^b^ *P* < 0.01 compared with the Normal group; ^d^ *P* < 0.01 compared with the Model group. The number of animals at 8 weeks is *n*_Normal_ = 8; *n*_Model_ = 7; *n*_Control A_ = 8; *n*_HXHT-M_ = 8. HXHT, Huoxue Huatan Decoction; I/R, ischemia/reperfusion; mtDNA, mitochondrial DNA.

The contents of active ingredients were 12.66% for tanshinone IIA, 9.25% for cryptotanshinone, 34.51% for salvianolic acid B, 5.30% for salvianolic acid A sodium, 1.64% for rosmarinic acid, 0.66% for protocatechuic aldehyde, 1.02% for ginsenoside Rg1, 0.90% for ginsenoside Rb1, 0.20% for notoginsenoside R1, 0.12% for ginsenoside Re, 25.81% for *G. biloba* total flavonols, 7.01% for *G. biloba* total lactones, 0.29% for the total saponins of *Z. jujuba* Mill., 0.10% for calycosin glucoside, and 0.10% for astragaloside IV (Lin, 2015). All of the components were tested by HPLC (Agilent 1100, Agilent Technologies, Inc., CA, USA). The conditions for the measurement of all of the components and HPLC spectra are shown in **Figures 1–23** in Part 1 of the **Supplementary Materials**. As positive controls, we used a traditional Chinese medicine (Danlou tablets, 0.3 g/tablet, product batch number: 20140605, Jilin Cornell Pharmaceutical Co., Ltd.) and a Western medicine (Atorvastatin calcium tablets, 20 mg/tablet, product batch number: J28283, sub-packaging batch number: J70319, Pfizer Pharmaceuticals LLC, Pfizer Pharmaceutical Co., Ltd., imported sub-packages).

### Wistar Rats

Male SPF Wistar rats (*N* = 168, weight 140 ± 10 g) were provided by Beijing Vital River Laboratory Animal Technology Co., Ltd., production license No. SCXK (Beijing) 2012-0001. The research protocol was approved by the Animal Ethics Committee of Guang’anmen Hospital of China Academy of Chinese Medicine (No. 2015EC035-02). In accordance with the Guide for the Care and Use of Laboratory Animals of the National Institutes of Health (Bethesda, MD, USA), they were raised indoors by the Animal Feeding Center of Guang’anmen Hospital, China Academy of Chinese Medical Sciences, at a constant temperature and with good ventilation, and they were fed with standard feed, with free access to food and water.

### Preparation of High-Fat Feed

The high-fat feed contained 78.8% of ordinary feed, 1% of cholesterol, 10% of yolk powder, 10% of lard, and 0.2% of bile salt (People’s Republic of China Ministry of Health, 2003). The nutritional composition of high-fat feed, provided by Beijing Huafukang Biotechnology Co., Ltd., is shown in **Table 2**.

**Table 2:** Nutrient composition of high-fat feed.

Nutrient composition	Ordinary feed (%)	High-fat feed (%)
Crude protein	20.16	18.89
Crude fat	4.24	19.34
Crude fiber	4.48	3.53
Coarse ash	6.04	4.76
Moisture	10	8.39
Calcium	1.29	1.02
Phosphorus	0.83	0.65

### Establishment of the I/R Injury Model in Hyperlipidemic Animals

After 3 days of quarantine, Wistar rats were randomly divided into the following seven groups (*n* = 24 rats per group): (i) healthy control group (Normal), (ii) hyperlipidemia model group (Model), (iii) Western medicine (Atorvastatin calcium tablets, 3.33 mg/kg) positive control group (Control A), (iv) traditional Chinese medicine (Danlou tablets, 0.75 g/kg) positive control group (Control B), (v) high-dose HXHT (3.6 g/kg, equivalent to 20.06 g/kg of the original medicinal material) group (HXHT-H), (vi) medium-dose HXHT (1.8 g/kg, equivalent to 10.03 g/kg of the original medicinal material) group (HXHT-M), and (vii) low-dose HXHT (0.9 g/kg, equivalent to 5.02 g/kg of the original medicinal material) group (HXHT-L). The calculation was based on the body surface area of humans and rats. HXHT was suspended in a 0.5% sodium carboxymethylcellulose aqueous solution (carboxymethyl cellulose nanoparticles, size 300–800 mpa·s, batch number: 20081023, manufacturer Sinopharm Chemical Reagent Co., Ltd.).

The Normal group was fed with ordinary feed, and the other groups were fed with high-fat feed. The rats were kept indoors with four animals per cage, at a constant temperature and under good ventilation, and they were fed with standard feed, with free access to food and water. After 4 weeks of feeding, the serum TG and LDL levels in all six high-fat groups were significantly increased compared with the Normal group. The Model group and the five treatment groups were appropriately adjusted according to the statistical results of cholesterol (CHO) and LDL levels, so that the mean values were as similar as possible. After these initial 4 weeks (*t* = 0), rats from the treatment groups were administered once a day a volume of 10 ml/kg for 4 or 8 weeks. The Normal and Model groups were simultaneously treated with an equal amount of 0.5% sodium carboxymethyl cellulose aqueous solution (10 ml/kg). Blood samples were collected from the eyeballs at the 4th, 6th, and 8th weeks to analyze biochemical indicators of blood lipids.

To establish the I/R model, the left anterior descending coronary artery of rats was ligated for 40 min (Liu et al., 2011; Du et al., 2014) and the blood flow was restored for 2 h, at the 4th and 8th weeks. The specific method was as follows.

① After weighing the rats, they were anesthetized with 10% chloral hydrate (330 mg/kg) (product batch number: 20130201, Sinopharm Chemical Reagent Co., Ltd.) by intraperitoneal injection. The rats were fixed on the operating table, the skin was prepared for disinfection, the tracheal tube was inserted, and the physiological signal acquisition system was connected.

② After tracheal intubation, the small animal ventilator was connected with a ventilator frequency of 70 breaths/min, a tidal volume of 8 ml, and a respiratory ratio of 1:3 for assisted respiration.

③ The third rib was cut at 0.5 cm from the left sternal border, the musculature was dissected, the thorax was opened with an eyelid opener to expose the heart, the needle was inserted 2−3 mm from the lower edge of the left atrial appendage with a 3/8 round needle pierced with a 4-0 suture and hooked around the main trunk of the left anterior descending coronary artery, and the needle was withdrawn from the pulmonary conus. A polyethylene tube was cut and placed between the two sutures, the sutures were tightened to form a closed loop, and reperfusion occurred by cutting the sutures 40 min later.

④ Successful criteria of model establishment were the following: the ST segment arch was elevated after ligation; the color of the ligation site was grayish or cyanotic; the ST segment regressed or was elevated after reperfusion; or the color of the ligation site changed from grayish to normal bright red.

⑤ During the whole process of myocardial I/R, a PowerLab physiological signal acquisition system was used to monitor electrocardiograms (ECGs) intermittently, and ECGs were continuously recorded before and after coronary artery ligation and after reperfusion. ST elevation was closely observed and recorded at the same time.

### Detection of Biochemical Indicators of Blood Lipids in Rats

Blood samples were collected from the retro-orbital venous plexus of the rats before treatment (at *t* = 0) and after 4, 6, and 8 weeks. Blood samples were stored at room temperature for 30 min and centrifuged at 3,000 rpm for 15 min to obtain serum. Four biochemical indicators of blood lipids were measured. The detection of CHO was performed using an enzymatic method (batch number: AUZ1433); the GPO-POD method was used for detection of TG (batch number: OSR61118E); high-density lipoprotein-cholesterol (HDL-C) levels were measured by an enzymatic method (batch number: OSR6287); and LDL-C levels were measured using an LDL-C enzymatic colorimetric assay kit (batch number: OSR6283), These kits were provided by Beckman Coulter Laboratories (Suzhou) Co., Ltd.

### Detection of CK-MB and LDH

Hyperlipidemic rats were anesthetized with 10% chloral hydrate (280 mg/kg) after 4 and 8 weeks, the skin was prepared, the trachea was intubated and connected to a ventilator, the chest was opened, the left coronary artery was ligated with a 4-0 sterile suture for 40 min, and perfusion was restored, while performing ECG monitoring. After 2 h of reperfusion, blood was collected from the intraocular canthus, stored for 30 min at room temperature, and centrifuged at 3,000 rpm for 15 min to obtain serum. The serum was diluted 20 times with purified water, and the creatine kinase-MB (CK-MB) and lactate dehydrogenase (LDH) levels were measured with an AU640 automatic biochemical analyzer. LDH was detected with an LDH lactate substrate assay kit (batch number: AUZ1443, Beckman Coulter Laboratories (Suzhou) Co., Ltd.); CK-MB was detected with a CK-MB enzymatic immunosuppression test kit (batch number: OSR61155, Beckman Coulter Laboratories (Suzhou) Co., Ltd.).

### Cardiac Function

After 4 or 8 weeks, the left coronary artery was ligated for 40 min, and reperfusion was allowed for 2 h. The rats were anesthetized with 10% chloral hydrate (280 mg/kg), prepared for skin disinfection, and fixed on the back. The left ventricular ejection fraction (LVEF), left ventricular end-diastolic inner diameter (LVIDd), left ventricular end-systolic inner diameter (LVIDs), and other cardiac function parameters were measured by DW-350 B-mode echocardiography (Dawei Electronic Equipment Co. Ltd., Xuzhou, JS, CHN).

### Calculation of Infarct Area and Ischemic Area

After 4 or 8 weeks, hyperlipidemic rats were anesthetized with 10% chloral hydrate (280 mg/kg), the left coronary artery was ligated for 40 min, and reperfusion was allowed for 2 h. The thoracic cavity was opened, the heart was exposed, the left anterior branch of the coronary artery was re-ligated along the suture, and 0.5% Evans blue (Yang et al., 2009) was intravenously injected in the left lung. The heart was stained blue about 2 min later. The heart was removed, rinsed with normal saline, frozen at −20°C, stained, and photographed using TTC. For statistical analysis, the Evans blue-stained blue area was considered as the normal area, the TTC-stained red area as the ischemic area, and the grayish white area as the infarct area. Photographs were taken with Canon PowerShot A2600 (after 4 weeks) and L2035AW Pioneer (after 8 weeks) cameras, and the myocardial ischemic, infarct, and global areas were analyzed with Image-Pro Plus 6.0 software. The ratio of the ischemic area to the global area and the ratio of the infarct area to the global area were calculated.

### Standard Operating Procedures for Pathological Staining

After 8 weeks, rats in the Normal, Model, Control A, and HXHT-M groups with better pharmacodynamic results were selected for histomorphological and molecular biological mechanism studies based on the previous experimental results. Rats in each group were subjected to myocardial ischemia for 40 min. After 2 h of reperfusion, they were anesthetized with 10% chloral hydrate mixture (280 mg/kg) and thoracotomy was performed. The hearts of the rats were removed and placed on ice, and 3 × 3 mm sized samples from the apical site were rapidly taken and fixed with 3% glutaraldehyde for sectioning. The mitochondrial ultramicrostructure in the infarct tissue was observed by transmission electron microscopy, with the assistance of the Peking University School. The 0.3 cm of myocardium below the ligature was fixed with 4% paraformaldehyde for He staining (Chen et al., 2018), another ~0.3 cm of myocardium was rapidly frozen for Oil Red O staining (Wen et al., 2019), and the rest was stored in a −80°C freezer.

### Detection of Oxidative Stress Indicators

After 8 weeks, after myocardial ischemia for 40 min and reperfusion for 2 h, and after anesthesia with 10% chloral hydrate (280 mg/kg), about 5 ml of blood was collected from the abdominal aorta. The artery was immediately clamped with the hemostatic forceps. The blood was allowed to stand for 40 min and centrifuged at 3,500 rpm for 10 min, and the serum was separated and stored in a −80°C freezer until analysis. The myocardial tissue samples were collected as described in the part of Standard Operating Procedures for Pathological Staining. The contents of malondialdehyde (MDA), total superoxide dismutase (T-SOD), CuZn-superoxide dismutase (CuZn-SOD), and glutathione peroxidase (GSH-Px) in rat serum and reactive oxygen species (ROS), succinate dehydrogenase (SDH), and cytochrome c oxidase (COX) in myocardial tissue were measured following the manufacturers’ instructions of the respective kits (MDA kit, batch number: 20150118, T-SOD kit, batch number: 20150312, Nanjing Jiancheng Biotechnology Co., Ltd.; CuZn-SOD kit, batch number: 20150204, Shanghai Youniko Co., Ltd.; GSH-Px kit, batch number: 20150206, Shanghai Youniko Co., Ltd.; ROS kit, batch number: GMS10096.2, Shanghai Jiemei Biological Gene Pharmaceutical Technology Co., Ltd.; SDH kit, batch number: 20150116, Nanjing Jiancheng Biotechnology Co., Ltd.; and COX kit, batch number: GMS10014.3.2, Shanghai Jiemei Biological Gene Pharmaceutical Technology Co., Ltd.).

### Detection of Protein, mRNA, and mtDNA

The protein expression levels of peroxisome proliferator-activated receptor-gamma coactivator 1 alpha (PGC-1α), peroxisome proliferator-activated receptor alpha (PPARα), nuclear respiratory factor 1 (NRF1), and mitochondrial transcription factor A (mtTFA) were measured by Western blot (Chen et al., 2018). The specific methods and steps are shown in Part 2 of the Supplementary Materials. The following antibodies were used: anti-PGC-1α (batch number: ab54481), anti-PPARα (batch number: ab8934), anti-NRF1 (batch number: ab175932), and anti-mtTFA (mitochondrial marker, batch number: ab131607) were provided by Abcam (Cambridge, UK); anti-β-actin (batch number: TA-09) was supplied by Beijing Zhongshan Jinqiao Biotechnology Co., Ltd. (Beijing, China). The mRNA expression levels of PGC-1α, PPARα, NRF1, and mtTFA were measured by real-time PCR (RT-PCR) (Mocker et al., 2019). The specific methods and steps are shown in Part 3 of the Supplementary Materials. Mitochondrial DNA (mtDNA) was detected using the PCR-fluorescent probe method (Liu et al., 2016). The specific methods and steps are shown in Part 4 of the **Supplementary Materials**.

### Statistical Analysis

SPSS 17.0 software was used for statistical analysis. The data are expressed as mean ± standard deviation $\left(\bar{x}\pm s\right)$. One-way analysis of variance (ANOVA) or the Student’s *t*-test was used for comparison between groups. Statistical results were considered to be statistically significant at *P* < 0.05. The area of myocardial infarction was analyzed with Image-Pro Plus 6.0 software, and the results were statistically analyzed with SPSS 17.0 software.

## Results

### Observation of Physical Signs

The body weight of rats in the Model group and the treatment groups was higher than that of rats in the Normal group, but this difference was not statistically significant. No abnormalities were found in mental status, physical activity, respiration, coat color, facial features, genitalia, food intake, urine, and feces.

### CHO Analysis

After 4 weeks of feeding with the high-fat diet (at *t* = 0), CHO levels were significantly higher in the Model group and the treatment groups than in the Normal group (*P* < 0.01), confirming the hyperlipidemic rat model was established successfully. The CHO serum levels were not significantly reduced in the Control A and B groups compared with the Model group during treatment (*P* > 0.05), although a decreasing trend was observed. HXHT-H could reduce CHO serum levels to different extents after 4, 6, and 8 weeks; after 6 and 8 weeks, the difference between the HXHT-H group and the Model group was statistically significant (*P* < 0.05 or *P* < 0.01). After 8 weeks, the HXHT-H, HXHT-M, and HXHT-L groups showed reductions in CHO levels of 30%, 21%, and 16%, respectively (**Figure 1**). In conclusion, HXHT significantly reduced CHO levels compared with Controls A and B.

### TG Analysis

After 4 weeks of high-fat feed (at *t* = 0), the TG serum levels were significantly higher in the Model group than in the Normal group (*P* < 0.01). The TG levels in the treatment groups were lower than in the Model group, but this difference was not statistically significant (*P* > 0.05). After 6 weeks of treatment, TG levels in the treatment groups were significantly lower. TG levels in the Control A and B groups had decreased by 21% and 18%, respectively (*P* < 0.01). TG levels in the HXHT-H, HXHT-M, and HXHT-L groups had decreased by 28% (*P* < 0.01), 20% (*P* < 0.01), and 10% (*P* < 0.05), respectively. In all of the treatment groups except for HXHT-H group, TG levels were lower after 8 weeks than after 6 weeks (*P* < 0.01; **Figure 2**). HXHT significantly reduced TG levels compared with Controls A and B, and there was no significant difference among the three HXHT groups.

### HDL-C Analysis

After 4, 6, and 8 weeks, HDL-C levels were significantly higher in the Model group than in the Normal group (*P* < 0.01). In all three HXHT groups, after 8 weeks, HDL-C levels were significantly lower than in the Model group (*P* < 0.05), and no significant changes were observed among the other groups (**Figure 3**). The reduction of HDL-C levels after 8 weeks of HXHT administration was significantly stronger than that in the Control A and Control B groups.

### LDL-C Analysis

Before treatment (at *t* = 0) and after 4, 6, and 8 weeks, LDL-C levels were increased to different extents in the Model group and the treatment groups compared with the Normal group (*P* < 0.05 or *P* < 0.01), confirming the hyperlipidemic rat model was established successfully.

LDL-C levels in all of the treatment groups began to decrease after 4 weeks compared with the Model group. After 8 weeks, LDL-C levels were significantly decreased in the Control A group (*P* < 0.05), while LDL-C levels in the Control B group exhibited no significant change (*P* > 0.05). LDL-C levels in the HXHT-H, HXHT-M, and HXHT-L groups had decreased by 47% (*P* < 0.01), 30% (*P* < 0.01), and 16% (*P* < 0.05), respectively. HXHT and Control A significantly reduced LDL-C levels compared with Control B after 8 weeks of administration (**Figure 4**).

### CK-MB and LDH Analysis

After 4 and 8 weeks, CK-MB levels after I/R injury were significantly higher in the Model group than in the Normal group (*P* < 0.05 or *P* < 0.01). CK-MB levels were significantly lower in the Control A, HXHT-H, and HXHT-M groups than in the Model group (*P* < 0.05 or *P* < 0.01). No significant differences were observed for other groups (*P* > 0.05; **Figure 5**). After 4 and 8 weeks, LDH serum levels after I/R injury were significantly higher in the Model group than in the Normal group (*P* < 0.01). LDH levels were significantly lower in the Control A, HXHT-H, and HXHT-M groups than in the Model group (*P* < 0.05 or *P* < 0.01). No significant changes were observed for the remaining groups (*P* > 0.05; **Figure 6**). HXHT and Control A controlled CK-MB and LDH levels significantly better than Control B after 4 and 8 weeks of administration.

### Analysis of Cardiac Function

After 4 weeks and I/R, no significant changes in LVEF, LVIDd, and LVIDs were observed in any group (**Figures 7** **–** **10**). After 8 weeks and 2 h of I/R, the LVEF, LVIDd, and LVIDs of rats in the Model group were significantly lower than in the Normal group (P < 0.05); the cardiac function of rats in all of the treatment groups was significantly better than in the Model group (P < 0.05 or P < 0.01). HXHT, Control A, and Control B significantly improved cardiac function, especially HXHT-M.

### Infarct Size and Ischemic Area Analysis

After 4 weeks, the area of myocardial infarction was larger in the Model group than in the Normal group, and the myocardial infarct size was reduced to different extents in the treatment groups compared with the Model group. The reduction in the area of myocardial infarction was more significantly reduced in the HXHT-M group (*P* = 0.057) than in the Control A and Control B groups. After 8 weeks, the area of myocardial infarction was significantly larger in the Model group than in the Normal group (*P* < 0.05). The area of myocardial infarction was significantly smaller in the treatment groups than in the Model group (*P* < 0.05 or *P* < 0.01; **Figure 11**).

After 4 weeks, the ischemic area of the Model group was slightly increased compared with the Normal group, but this difference was not statistically significant. The ischemic area in the Control A and HXHT-H groups was significantly smaller than in the Model group (*P* < 0.05), and was larger than that in the Control B group. After 8 weeks, no significant difference was observed between any groups. The results of our analysis of infarct size and ischemic area are presented in **Figure 11**.

### HE Staining Analysis

After 8 weeks and I/R injury, HE staining of myocardial tissue revealed the following.

Normal group: Local myocardial cells had small focal necrosis, the arrangement of local myocardial fibers was disordered, wavy, or broken, and granular degeneration and vacuole degeneration could be observed locally. Myocardial capillaries were dilated and hyperemia was observed; inflammatory cell infiltration was observed under the epicardium.

Model group: Local myocardial cells had small focal necrosis, and the arrangement of local myocardial fibers was disordered, wavy, or broken, with varying degrees of granular degeneration and vacuolar degeneration. The myocardial interstitium was visibly widened, the myocardial capillaries were dilated, and congestion was obvious. Inflammatory cell infiltration was observed in myocardial interstitium, perivasculature, and epicardium, which was more pronounced than in the Normal group.

Control A group: Local myocardial cells had small focal necrosis, and the arrangement of local myocardial fibers was disordered, wavy, or broken, with a low degree of cell degeneration and varying degrees of granular degeneration and vacuolar degeneration. Capillary congestion, myocardial interstitial widening, and inflammatory cell infiltration were also observed, but these were milder than in the Model group.

HXHT-M group: Mild loose edema of myocardial cells was observed, with local small focal necrosis, occasional wavy myocardial fibers, slight granular degeneration and vacuolar degeneration, and inflammatory cell infiltration, which were milder than in the Model group (**Figure 12**).

### Oil Red O Staining Analysis

After 8 weeks and I/R, the results of Oil Red O staining of myocardial tissue revealed the following:

Normal group: Dark red lipid droplets were occasionally observed locally and scattered in cardiomyocytes. Occasionally, the light brown lipid droplets were connected into sheets, and light brown or tan miscellaneous droplets were observed overall.

Model group: Dark red lipid droplets were often observed scattered in cardiomyocytes, which also contained larger lipid droplets. Light reddish brown lipid droplets at the epicardium were connected into sheets, and brownish red or tan miscellaneous granules were observed overall.

Control A group: Dark red lipid droplets were occasionally scattered in cardiomyocytes, with fewer lipid droplets. The whole body was light brown, but occasionally brown red was found in the folds.

HXHT-M group: Dark red lipid droplets were occasionally scattered in cardiomyocytes, with fewer lipid droplets, and the whole body was light brown. The epicardium was reddish brown, and occasionally brown red was found in the folds.

Oil Red O staining showed that there were more lipid droplets in the Model group, while there were fewer lipid droplets in the Control A and HXHT-M groups (**Figure 13**).

### Analysis of Mitochondrial Ultrastructure

After 8 weeks and I/R injury, the mitochondrial ultrastructure analysis revealed the following.

Normal group: The mitochondrial ultrastructure was relatively clear and the membrane was relatively intact, but the mitochondrial cristae were vague and disordered, fused, and damaged. Some mitochondrial cristae disappeared or vacuolized, and the ruptured membranes rarely formed vesicles or vacuoles. Occasionally, disordered intercalated discs and poor continuity were observed.

Model group: Mitochondria were significantly swollen, the inner and outer membranes were obviously damaged, or even broken and dissolved, and the contents spilled. Mitochondrial cristae were vague and disordered, and some mitochondrial cristae disappeared or vacuolized. The mitochondrial matrix was blurred, and fragmented mitochondria were observed. Myofibrils were uneven in thickness, the sarcomere structure was unclear, and some myofilaments were damaged and dissolved. Some disordered intercalated discs and poor continuity were observed. Most of the myofilaments were dissolved, and the Z-line was significantly thickened extending toward the I-band.

Control A group: The mitochondrial membrane was thinned or even ruptured. The mitochondrial ridges were reduced and the ridges were blurred. Some mitochondrial cristae disappeared or vacuolized. Mitochondria were swollen, partial rhomboid cristae and longitudinal cristae were observed, and giant myocardial mitochondria, vesicles, or vacuoles formed from ruptured membranes. The intercalated discs were neat and continuous.

HXHT-M group: The ultrastructure of rat myocardial mitochondria was clear, the membrane was intact, the mitochondrial cristae were dense, the mitochondrial matrix was clear, and occasional mitochondrial cristae were blurred. The intercalated discs were neat and continuous. Mitochondria were distributed in bands and arranged neatly. The sarcomeres were well arranged.

In the Model group, mitochondria were swollen. The mitochondrial membrane was ruptured. Mitochondrial cristae fused, disappeared, or broke. These phenomena were more serious than in other groups (**Figure 14**).

### Analysis of Oxidative Stress

After 8 weeks and I/R injury, MDA serum levels were significantly higher in the Model group than in the Normal group (*P* < 0.01). MDA serum levels were lower in the Control A and HXHT-M groups than in the Model group; for the HXHT-M group, this difference was statistically significant (*P* < 0.05).

T-SOD, CuZn-SOD, and GSH-Px levels were significantly lower in the Model group than in the Normal group (*P* < 0.05 or *P* < 0.01). T-SOD, CuZn-SOD, and GSH-Px levels were higher in the Control A and HXHT-M groups than in the Model group; for T-SOD in the HXHT-M group, this difference was statistically significant (*P* < 0.01).

The SDH levels in myocardial tissue after myocardial I/R were significantly lower in the Model group than in the Normal group (*P* < 0.05), and the SDH levels in the HXHT-M group were significantly higher than in the Model groups (*P* < 0.05). No significant differences in ROS and COX levels were observed between the groups (**Figure 15**).

### mRNA and Protein Expression Levels of PGC-1α, PPARα, NRF1, and mtTFA

After 8 weeks and I/R injury, the mRNA and protein expression levels of PGC-1α, PPARα, NRF1, and mtTFA were significantly lower in the Model group than in the Normal group (*P* < 0.01). The mRNA expression levels of PGC-1α, PPARα, NRF1, and mtTFA were significantly increased in the Control A and HXHT-M groups compared with the Model group (*P* < 0.05; **Figure 16**). The protein expression levels of PGC-1α, NRF1, and mtTFA were significantly increased in the Control A and HXHT-M groups compared with the Model group (*P* < 0.05 or *P* < 0.01; **Figure 17**). The protein expression levels of PPARα were significantly increased in the HXHT-M group compared with the Model group (*P* < 0.05; **Figure 17**), but the increase was not significant in the Control A group.

### Analysis of mtDNA Copy Number

After 8 weeks and I/R injury, the mtDNA copy number was significantly lower in the Model group than in the Normal group (*P* < 0.01), and it was significantly increased in the HXHT-M group compared with the Model group, while it was further decreased in the Control A group (*P* < 0.01; **Figure 18**).

## Discussion

Elevated blood lipids are one of the main pathogenic factors of atherosclerosis (Song, 2003). LDL-C has a direct relationship with atherosclerotic cardiovascular disease and is positively correlated with the incidence of the disease. The main goal of preventing and treating atherosclerotic cardiovascular disease is to reduce abnormal blood lipids, especially LDL-C. Therefore, here we conducted an in-depth study of the dynamic continuous and dose-effect relation of the effects of HXHT on lipid metabolism in hyperlipidemic rats. Our results showed that a high-fat diet could significantly increase the levels of CHO, TG, HDL-C, and LDL-C in Wistar rats. HXHT could significantly reduce blood lipids to different extents after 4, 6, and 8 weeks of administration, and the effects of high-dose HXHT were the most significant. HXHT can significantly reduce CHO, TG, LDL-C, and HDL-C after 4, 6, and 8 weeks of administration. HXHT was superior to the positive control drugs, and it had no significant effect on body weight. *S. miltiorrhiza* Bunge, *P. notoginseng* (Burkill) F.H. Chen, *T. kirilowii* Maxim., and *A. macrostemon* Bunge, which are ingredients of HXHT, have previously been reported to have hypolipidemic effects. For example, salvianolic acid B could reduce blood glucose, increase insulin resistance, reduce CHO, LDL-C, free fatty acid, liver glycogen, and muscle glycogen, and increase HDL-C in diabetic model rats (Huang et al., 2015). *P. notoginseng* (Burkill) F.H. Chen could significantly reduce CHO, TG, and LDL-C and increase HDL-C in the treatment of patients with hyperlipidemia (Si, 2015). *T. kirilowii* Maxim. and *A. macrostemon* Bunge could significantly reduce TC, TG, LDL, and arteriosclerosis index (*P* < 0.01). The combination of the two had a synergistic effect and a better lipid-lowering effect (He, 2002).

A high-fat and low-sugar diet did not affect non-ischemic left ventricular function, but it could aggravate myocardial injury and enhance the mortality caused by pump failure and ventricular arrhythmia during myocardial I/R in rats, which might be closely related to myocardial mitochondrial fission and fusion, calcium channel, and myocardial capacity metabolism (Liu and Lloyd, 2013; Liu et al., 2014). The effect of HXHT on myocardial I/R injury in hyperlipidemic rats was studied. The results showed that after 8 weeks and 12 weeks of feeding, the myocardial enzyme levels, cardiac function, and the myocardial infarction area of the hyperlipidemia model group were significantly higher, worse, and larger than those of the Normal control group, respectively. A high-fat diet could aggravate myocardial I/R injury in rats. HXHT could prevent myocardial I/R injury in hyperlipidemic rats after 4 weeks of administration, and significantly reduce myocardial enzyme levels. After 8 weeks of administration, HXHT could significantly reduce the myocardial infarction area and improve cardiac function.

The histomorphological study showed that myocardial cell focal necrosis, myocardial vascular congestion, inflammatory cell infiltration, and lipid droplet accumulation were more serious in the hyperlipidemia model than in the other groups, while in the HXHT groups and the Control A group, these were lighter than in the hyperlipidemia model group. On the one hand, HXHT might reduce the infiltration of inflammatory cells due to its lipid-lowering effects; on the other hand, the components of HXHT may have anti-inflammatory effects. Tanshinone and salvianolic acid could promote blood circulation, dilate blood vessels, scavenge free radicals, and protect mitochondria, and they have anticoagulation and anti-inflammatory effects (Adams et al., 2006). Cryptotanshinone, which is also present in HXHT, could downregulate the expression of pro-inflammatory cytokines; cryptotanshinone and 15,16-dihydrotanshinone could inhibit the activities of nuclear transcription factor-κB and activator protein (Jeon et al., 2008). There are many ways to inhibit atherosclerosis. Salvianolic acid B and ginkgolide mainly act on inflammatory cytokines, *P. notoginseng* glycosides act on adhesion factors, and tanshinone acts on growth factors (Li et al., 2007).

Oxygen free radicals, calcium overload, and the inflammatory response are directly or indirectly related to mitochondria, which function as intracellular effectors and are protected by ischemic preconditioning. Mitochondria, as an important organelle in the regulation of energy metabolism in cells, participate in myocardial I/R injury through various pathways. The results of the present study showed that the mitochondria in the hyperlipidemia model group were swollen and the mitochondrial membrane was ruptured. HXHT could protect the morphology of mitochondria upon I/R. Excessive fat intake increases oxygen free radical levels. Free radicals cause lipid peroxidation of cell membranes, causing mitochondrial dysfunction, which in turn affects the tricarboxylic acid cycle and results in dysfunction of the myocardium. The results of this study showed that the activities of MDA, T-SOD, CuZn-SOD, and GSH-PX in serum were significantly changed after myocardial I/R in the hyperlipidemia model group, and SDH levels in myocardial tissue were significantly decreased. HXHT could reduce the activity of MDA in serum, increase the activity of T-SOD, and increase the activity of SDH in myocardial tissue. It was speculated that HXHT might: (i) enhance the ability of rats to resist oxygen free radicals, (ii) prevent oxygen free radicals from invading mitochondrial membranes, and (iii) protect mitochondrial membranes, thereby protecting the function of mitochondria (Liu et al., 2014).

The results of this experimental study showed that HXHT may protect the myocardium through the PGC-1α–PPAR pathway and regulate mitochondrial biosynthesis through the PGC-1α–NRF1–mtTFA pathway. The two pathways coordinate to protect myocardial mitochondria during myocardial I/R. In the PGC-1α–PPARα pathway, PPARα could regulate myocardial energy and fat metabolism, participate in every step of myocardial utilization of fatty acids, and play a decisive role in myocardial energy generation (Barroso et al., 2013).

HXHT could increase the gene and protein expression levels of PGC-1α and downstream PPARα; these results were consistent with the lipid-lowering effects of HXHT. PPARα is mainly expressed in the liver and participates in lipid regulation, adipogenesis, and blood glucose control. PPARα affects lipid metabolism by regulating fatty acid oxidation. In addition, PPARα is expressed in various cells constituting the blood vessel wall, such as monocyte-macrophages, smooth muscle cells, and vascular endothelial cells. HXHT could promote the expression of PPARα. PPARα directly acts on the inner wall of the blood vessel, increases the antioxidant effect of the vascular wall, and plays a role in the protection of blood vessels. HXHT might regulate fatty acid β-oxidation to ensure energy supply after I/R and rapidly restore the myocardium. Previous studies on the PGC-1α–NRF1–mtTFA pathway have shown that high fat intake could reduce the mRNA and protein levels of the mitochondrial biogenesis-related genes PGC-1α, NRF1, and mtTFA (Keaney et al., 2003; Liu et al., 2014). HXHT could promote the mRNA and protein expression levels of PGC-1α, NRF1, and mtTFA in the myocardium of hyperlipidemic rats in preliminary experiments, and alleviate myocardial I/R injury. On the one hand, HXHT lowered blood lipids and reduced the damage of CHO and LDL-C to the vascular endothelium; on the other hand, it reduced oxygen free radical levels in mitochondria, and acted on other pathways through its effects on NRF1 and mtTFA. HXHT might increase the expression of mtDNA and promote mitochondrial biosynthesis by promoting the mRNA and protein expression of PGC-1α, NRF1, and mtTFA and increasing PGC-1α–PPARα pathway activity.

## Conclusions

The results of this study demonstrate that a high-fat diet affects myocardial mitochondria through two pathways: PGC-1α–PPARα and PGC-1α–NRF1–mtTFA. Myocardial fatty acid biosynthesis and oxidation affect mitochondrial function and aggravate I/R injury, as revealed by a comprehensive study of lipid metabolism, the myocardial enzyme spectrum, myocardial histomorphology, and mitochondrial biogenesis. HXHT could affect lipid metabolism and reduce the levels of CHO, TG, HDL-C, and LDL-C in hyperlipidemic rats in a dose-dependent manner, affect fatty acid β-oxidation through the PGC-1α–PPARα pathway, and protect against disturbances of mitochondrial energy metabolism. At the same time, HXHT could promote the mRNA expression and protein transcription of the mitochondrial biosynthesis-related genes of PGC-1α–NRF1–mtTFA, promote mtDNA synthesis, increase T-SOD levels, protect the structure and function of mitochondria, and defend against myocardial I/R injury in hyperlipidemic rats.

## Data Availability Statement

The raw data supporting the conclusions of this article will be made available by the authors, without undue reservation, to any qualified researcher.

## Ethics Statement

The animal study was reviewed and approved by Animal Ethics Committee of Guang’anmen Hospital of China Academy of Chinese Medicine.

## Author Contributions

All of the authors took part in the experiments. FL and Y-QT wrote the first draft of the manuscript. X-HH, L-LG, and B-JW revised the manuscript. H-WC, J-PL, and ZC revised sections of the manuscript. H-WC and JW drew article figures and tables. All authors contributed to the article and approved the submitted version.

## Funding

This study was supported by the National Major Scientific and Technological Special Project for “Significant New Drugs Development” (No. 2012ZX09102201), the National Natural Science Foundation of China (No. 81973836), the China Association for Science and Technology Youth Talent Project (No. 2017QNRC001), and the China Academy of Chinese Medical Sciences—Traditional Chinese Medicine Science and Technology Achievement Leading Project (No. ZZ13-ZD-04).

## Conflict of Interest

The authors declare that the research was conducted in the absence of any commercial or financial relationships that could be construed as a potential conflict of interest.
