# Supplementary material for: Huoxue Huatan Decoction Ameliorates Myocardial Ischemia/Reperfusion Injury in Hyperlipidemic Rats via PGC-1α–PPARα and PGC-1α–NRF1–mtTFA Pathways
Source: Front Pharmacol. 2020 Sep 15;11:546825. doi: 10.3389/fphar.2020.546825 (PMC7522555; doi:10.3389/fphar.2020.546825)
Supplement: Supplementary file 1 [file DataSheet_1.docx]

**Huoxue Huatan Decoction ameliorates myocardial ischemia/reperfusion injury in hyperlipidemic rats via PGC-1α–PPARα and PGC-1α–NRF1–mtTFA pathways**

Fei Lin^1^, Yu-Qing Tan^2,3^, Xuan-hui He^2^, Li-li Guo^2^, Ben-jun Wei^4^, Jun-ping Li^2^, Zhong Chen^2^, Heng-Wen Chen^2*^, Jie Wang^2*^

*1 Heart Center of Xinxiang Medical University, The First Affiliated Hospital of Xinxiang Medical University, Xinxiang, China*

*2 Department of Cardiology, Guang’anmen Hospital, China Academy of Chinese Medical Sciences, Beijing, China*

*3 Graduate School, Beijing University of Chinese Medicine，Beijing, China*

*4 Key Laboratory of Ministry of Education Department of Lanzhou Province and Dunhuang Medical Transformation, Gansu University of Chinese Medicine, Lanzhou, China*

Fei Lin and Yu-Qing Tan contributed equally to this work.

*Correspondence: The authors have contributions equally to this work.

Heng-Wen Chen, [chenhengwen@163.com](mailto:chenhengwen@163.com); Jie Wang, [jiewang1001@126.com](mailto:13051458913@163.com)

**Supplementary Materials**

**1 HPLC Spectrum of Active Ingredients of HXHT**

**1.1 The Content of Tanshinone IIA Determined by HPLC**

Chromatographic conditions: chromatographic column: Agilent TC-C18 (4.6×150 mm, 5 μm); mobile phase: acetonitrile (A)–0.02% phosphoric acid aqueous solution (B) gradient elution; the gradient elution ratio is shown in **Table 1**; flow rate: 1 mL/min; injection volume: 10 μL; column temperature: 20°C; detection wavelength: 270 nm.

TABLE 1︱Gradient elution ratio of the mobile phase in tanshinone IIA.

| Time (min) | 0 | 6 | 20 | 20.5 | 25 |
| --- | --- | --- | --- | --- | --- |
| A (%) | 61 | 61 | 90 | 61 | 61 |
| B (%) | 39 | 39 | 10 | 39 | 39 |

The HPLC spectra are shown in **Figure 1** and **Figure 2** for tanshinone IIA (*t* = 14.23 min) and cryptotanshinone (*t* = 10.00 min).


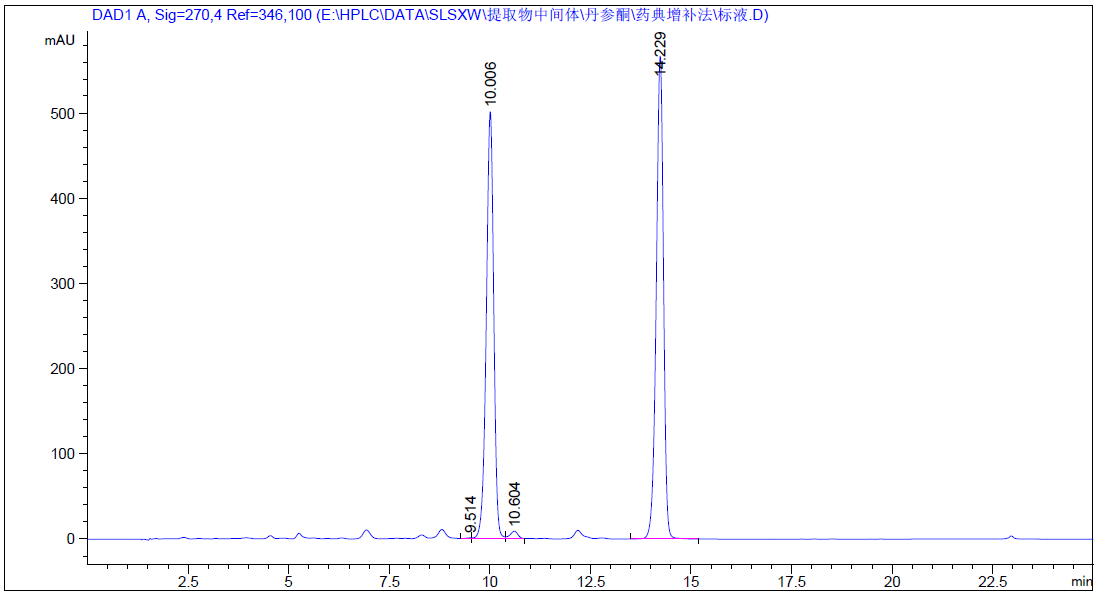


1

2

FIGURE 1︱HPLC spectrum of canshinone IIA reference substance.


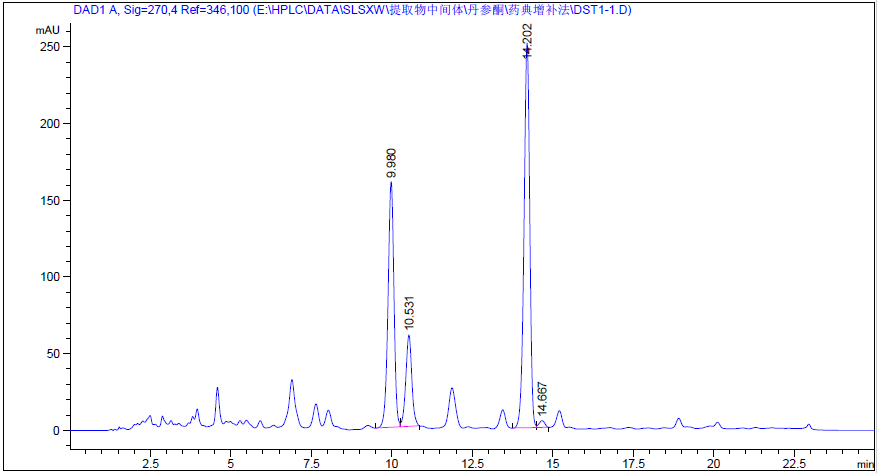


1

2

FIGURE 2︱HPLC spectrum of tanshinone IIA in HXHT extracts.

(Peak 1, cryptotanshinone; Peak 2, tanshinone IIA)

**1.2 The Content of Salvianolic Acid B Determined by HPLC**

Chromatographic conditions: chromatographic column: YMC ODS-H18 (4.6×150 mm, 5 μm); mobile phase: acetonitrile (A)–0.05% phosphoric acid aqueous solution (B) gradient elution; the gradient elution ratio is shown in **Table 2**; flow rate: 1.0 mL/min; injection volume: 10 μL; column temperature: 30°C; detection wavelength: 286 nm.

TABLE 2︱Gradient elution ratio of the mobile phase in salvianolic acid B.

| Time(min) | 0 | 15 | 30 | 40 | 50 |
| --- | --- | --- | --- | --- | --- |
| A (%) | 17 | 23 | 25 | 90 | 90 |
| B (%) | 83 | 77 | 75 | 10 | 10 |

The HPLC spectra are shown in **Figure 3** and **Figure 4** for salvianolic acid B (*t* = 18.10 min).


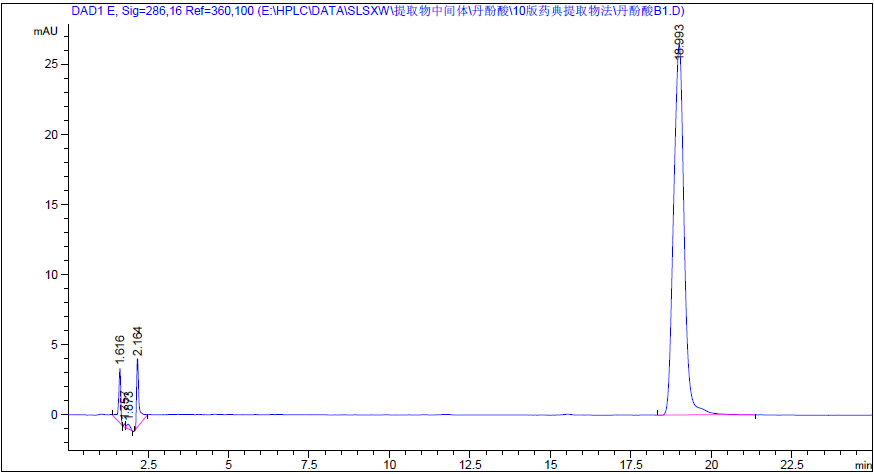


1

FIGURE 3︱HPLC spectrum of salvianolic acid B reference substance.


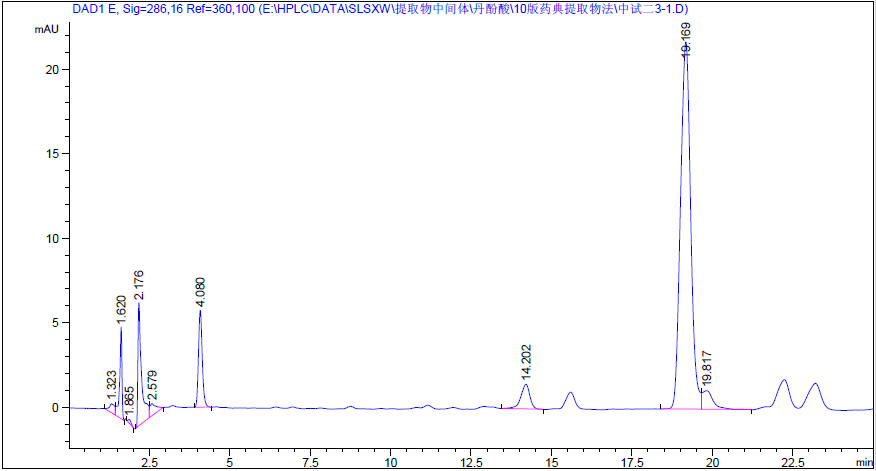


1

FIGURE 4︱HPLC spectrum of salvianolic acid B in HXHT extracts

(Peak 1, salvianolic acid B).

**1.3 Determination of Total Flavonoids in *Ginkgo biloba* L. Extract**

Chromatographic conditions: chromatographic column: Agilent SB-C18 (4.6×250 mm, 5 μm); mobile phase: methanol–0.4% phosphoric acid aqueous solution; flow rate: 1 mL/min; injection volume: 10 μL; column temperature: 25°C; detection wavelength: 360 nm. The total flavonoids were calculated as the sum of quercetin (*t* = 12.62 min), kaempferol (*t* = 22.32 min), and isorhamnetin (*t* = 25.37 min). The HPLC spectra are shown in **Figure 5** and **Figure 6**.


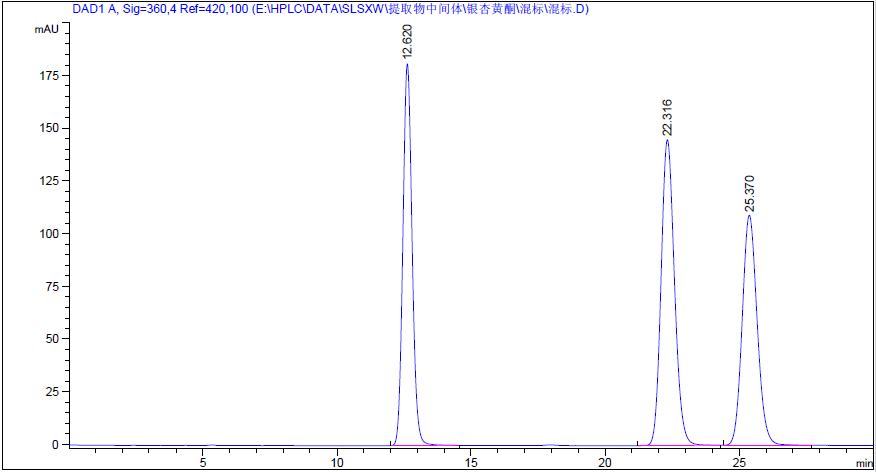


3

2

1

FIGURE 5︱HPLC spectrum of mixed reference substance of total flavonoids.


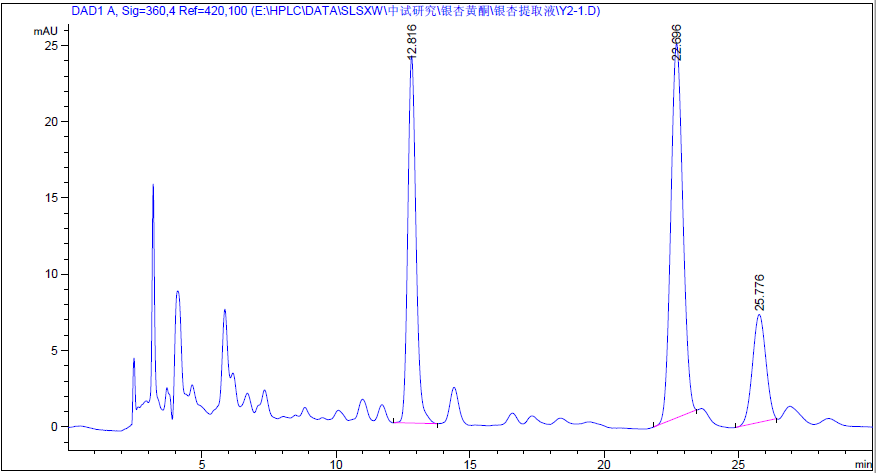


3

2

1

FIGURE 6︱HPLC spectrum of total flavonoids in HXHT extracts

(Peak 1, quercetin; Peak 2, kaempferol; Peak 3, isorhamnetin).

**1.4 Determination of Total Lactones in *Ginkgo biloba* L. Extract**

Chromatographic conditions: Chromatographic column: Agilent Zorbax XDB-C18 (4.6×150 mm, 5 μm); mobile phase: n-propanol–tetrahydrofuran–water (1-15-84); flow rate: 1 mL/min; injection volume: 10 ; column temperature: 25°C; ELSD detection: gas flow rate 3.5 L/min; temperature: 105°C. The total lactones of *Ginkgo biloba* L. were calculated as the sum of ginkgolides A (GA), ginkgolides B (GB), ginkgolides C (GC), and bilobalide (BB). The HPLC spectra are shown in **Figure 7** and **Figure 8**.


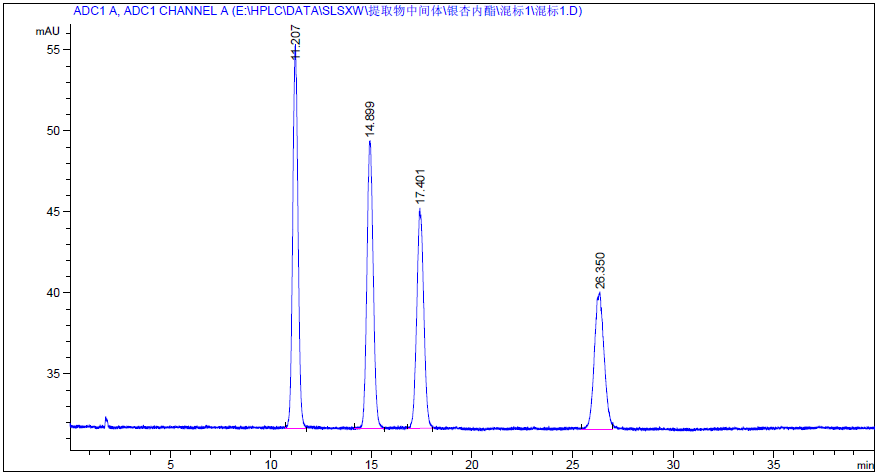


4

3

2

1

FIGURE 7︱HPLC spectrum of mixed reference substance of total lactones.


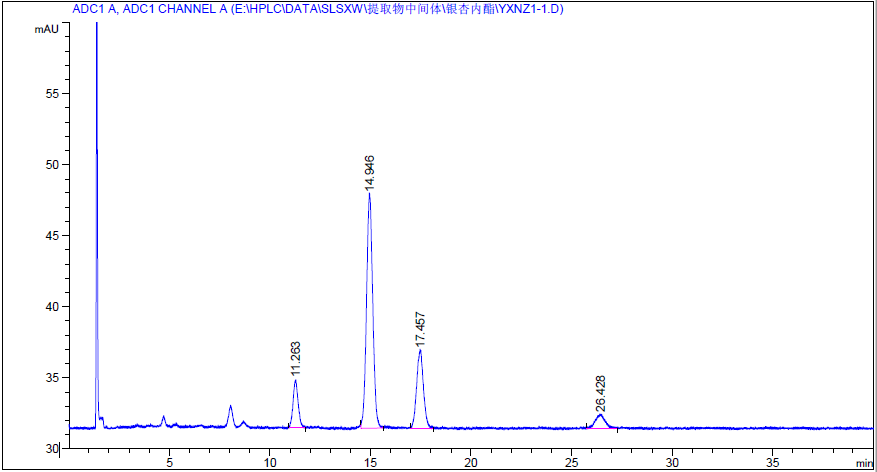


4

3

2

1

FIGURE 8︱HPLC spectrum of total lactones in HXHT extracts

(Peak 1, ginkgolide C; Peak 2, bilobalide; Peak 3, ginkgolide A; Peak 4, ginkgolide B).

**1.5 Determination of *Panax notoginseng* Saponins in the Mixed Extracts**

Chromatographic conditions: chromatographic column: Kromasil C18 (4.6×250 mm, 5 μm), mobile phase: acetonitrile (A)–water (B) gradient elution; the gradient elution ratio is shown in **Table 3**; flow rate: 1.5 mL/min; injection volume: 10 μL; column temperature: 25°C; detection wavelength: 203 nm. The HPLC spectra are shown in **Figure 9**, **Figure 10**, and **Figure 11**.

TABLE 3︱Gradient elution ratio of the mobile phase in *Panax notoginseng* saponins.

| Time(min) | | 0 | 20 | 45 | 55 | 60 |
| --- | --- | --- | --- | --- | --- | --- |
| A (%) | 20 | | 20 | 46 | 55 | 55 |
| B (%) | 80 | | 80 | 54 | 45 | 45 |


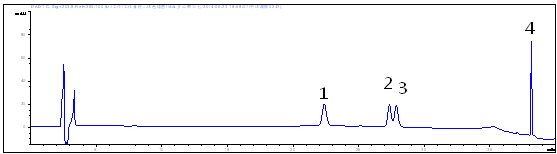


FIGURE 9︱HPLC spectrum of mixed reference substance of *Panax notoginseng* saponins.

FIGURE 10︱HPLC spectrum of *Panax notoginseng* saponins in HXHT extracts lacking *Panax notoginseng* (Burkill) F.H. Chen.


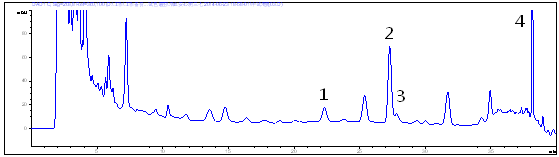


FIGURE 11︱HPLC spectrum of *Panax notoginseng* saponins in HXHT extracts (Peak 1, notoginsenoside R1; Peak 2, ginsenoside Rg1; Peak 3, ginsenoside Re; Peak 4, ginsenoside Rb1).

**1.6 Determination of the Total Saponins of *Ziziphus jujuba* Mill. in the Mixed Extracts**

Chromatographic conditions: chromatographic column: Agilent HC-C18 (4.6×250 mm, 5 μm), mobile phase: acetonitrile–0.05% phosphoric acid aqueous solution (36-64); flow rate: 1 mL/min; injection volume: 10 μL; column temperature: 25°C; detection wavelength: 204 nm. The HPLC spectra are shown in **Figure 12**, **Figure 13**, and **Figure 14**.


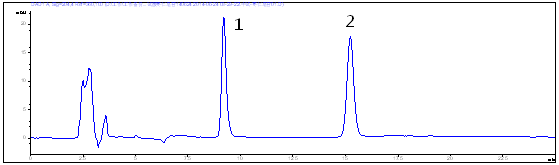


FIGURE 12︱HPLC spectrum of mixed reference substance of the total saponins of *Ziziphus jujuba* Mill.

FIGURE 13︱HPLC spectrum of the total saponins of *Ziziphus jujuba* Mill. in HXHT extracts lacking *Ziziphus jujuba* Mill.


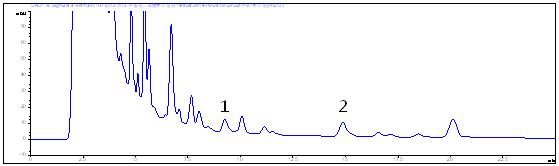


FIGURE 14︱HPLC spectrum of the total saponins of *Ziziphus jujuba* Mill. in HXHT extracts

(Peak 1, jujuboside A; Peak 2, jujuboside B).

**1.7 Determination of Spinosin in the Mixed Extracts**

Chromatographic conditions: chromatographic column: Agilent HC-C18 (4.6×250 mm, 5 μm), mobile phase: acetonitrile (A)–water (B) gradient elution; the gradient elution ratio is shown in **Table 4**; flow rate: 1 mL/min; injection volume: 10 μl; column temperature: 25 ℃; detection wavelength: 335 nm. The HPLC spectra are shown in **Figure 15**, **Figure 16**, and **Figure 17**.

TABLE 4︱Gradient elution ratio of the mobile phase in spinosyn.

| Time (min) | 0 | 10 | 16 | 22 | 30 |
| --- | --- | --- | --- | --- | --- |
| A (%) | 12 | 19 | 20 | 100 | 100 |
| B (%) | 88 | 81 | 80 | 0 | 0 |

1

FIGURE 15︱HPLC spectrum of spinosin reference substance.

FIGURE 16︱HPLC spectrum of spinosin in HXHT extracts lacking *Ziziphus jujuba* Mill.

1

FIGURE 17︱HPLC spectrum of spinosine in HXHT extracts

(Peak 1, spinosin).

**1.8 Determination of Astragaloside IV in the Mixed Extracts**

Chromatographic conditions: chromatographic column: Agilent TC-C18 (4.6 × 150 mm, 5 μm), mobile phase: acetonitrile–water (32-68); flow rate: 1 mL/min; injection volume: 10 μL; column temperature: 30°C; ELSD detection: air flow 2.8 L/min; temperature: 105°C. The HPLC spectra are shown in **Figure 18**, **Figure 19**, and **Figure 20**.

min

2.5

5

7.5

10

12.5

15

17.5

20

mAU

25

27.5

30

32.5

35

37.5

40

42.5

45

47.5

ADC1 A, ADC1 CHANNEL A (D:\??\????...\?????\??-????140722 2014-07-22 14-37-25\????.D)

1

FIGURE 18︱HPLC spectrum of astragaloside IV reference substance.

FIGURE 19︱HPLC spectrum of astragaloside IV in HXHT extracts lacking *Astragalus mongholicus* Bunge.

1

FIGURE 20︱HPLC spectrum of astragaloside IV in HXHT extracts

(Peak 1: astragaloside IV).

**1.9 Determination of Calycosin Glucoside in Mixed Extracts**

Chromatographic conditions: Chromatographic column: Agilent TC-C18 (4.6 × 150 mm, 5 μm); mobile phase: acetonitrile (A)–0.2% formic acid aqueous solution (B) gradient elution; the gradient elution ratio is shown in **Table 5**; flow rate: 1 mL/min; injection volume: 10 μL; column temperature: 25 °C; detection wavelength: 260 nm. The HPLC spectra are shown in **Figure 21**, **Figure 22**, and **Figure 23**.

TABLE 5︱Gradient elution ratio of the mobile phase in calycosin glucoside.

| Time (min) | 0 | 20 | 30 |
| --- | --- | --- | --- |
| A (%) | 20 | 40 | 40 |
| B (%) | 80 | 60 | 60 |

1

FIGURE 21︱HPLC spectrum of calycosin glucoside reference substance.

FIGURE 22︱HPLC spectrum of calycosin glucoside in HXHT extracts lacking *Astragalus mongholicus* Bunge.

1

FIGURE 23︱HPLC spectrum of calycosin glucoside in HXHT extracts

(Peak 1, calycosin glucoside).

**2 The mRNA Expression Levels of PGC-1α, PPARα, NRF1, and mtTFA Detected by RT-PCR**

**2.1 Preparation of Total RNA of Cardiomyocytes**

① Rat myocardial tissue sample (100 mg) was taken, 1 ml TRIzol was added, and after sonication samples were placed at room temperature for 10 minutes. Samples were centrifuged at 12,000 rpm for 10 minutes at 4 °C, and the supernatant was collected in a new tube. ② Pre-cooled chloroform (200 µl) was added, and samples were mixed well and placed at room temperature for 5 minutes. ③ Samples were centrifuged at 12,000 rpm for 15 minutes at 4°C, and the supernatant (about 500 µL) was collected in a new tube. ④ Pre-cooled isopropanol (500 µl) was added, and samples were placed at −20°C for 30 minutes and centrifuged at 12,000 rpm for 15 minutes at 4°C. The supernatant was discarded. ⑤ Pre-cooled 75% ethanol (800–1000 µl) was added, and samples were mixed well and centrifuged at 12,000 rpm for 10 minutes at 4°C. The supernatant was discarded, and this step was repeated. ⑥ Pellets were dried for 30 min, and when the RNA was translucent, 30 µl DEPC H_2_O was added, and RNA was dissolved in water at 55°C for 10 minutes. ⑦ After mixing, 1 µL was taken and the OD value (260 nm, 280 nm) was measured with NanoDrop 2000 (**Table 6**). ⑧ The remaining samples were stored at −80°C or reverse transcribed immediately.

TABLE 6︱RNA concentration and OD ratio (260/280) of each group.

|  | Nucleic acid conc. (µg/µL) | A260 | A280 | Ratio (260/280) |
| --- | --- | --- | --- | --- |
| Normal control | 1.22 | 30.41 | 15.22 | 2.00 |
| Model control | 1.70 | 42.52 | 21.03 | 2.02 |
| Control A | 1.28 | 32.03 | 15.96 | 2.01 |
| HXHT-M | 1.24 | 30.92 | 15.48 | 2.00 |

**2.2 RNA Reverse Transcription**

The first-strand cDNA was synthesized using the fast quant RT kit.

① The mixture was prepared according to the genomic DNA removal system in **Table 7**, mixed well, centrifuged, incubated at 42°C for 3 minutes, and then placed on ice.

TABLE 7︱gDNA removal reaction system.

| Composition | Usage amount |
| --- | --- |
| 5× gDNA Buffer | 2 μL |
| 50 ng–2 μg total RNA | 1 μL |
| RNase-free ddH_2_O | Add up to 10 μL |

② The mixed solution was prepared in accordance with the reverse transcription reaction system in **Table 8**.

TABLE 8︱Reverse transcription reaction system.

| Composition | Usage amount |
| --- | --- |
| 10× Fast RT Buffer | 2 μL |
| RT Enzyme Mix | 1 μL |
| FQ-RT Primer Mix | 2 μL |
| RNase-free ddH_2_O | Add up to 10 μL |

③ The gDNA-free samples from Step 1 were used as templates for the reverse transcription reaction and mixed well. ④ Samples were incubated at 42°C for 15 minutes. ⑤ After incubation at 95°C for 3 minutes, samples were put on ice. The obtained cDNA can be used for subsequent experiments or stored at low temperature.

**2.3 Real-time PCR Amplification**

The amplification reaction was performed on a 7900HT fluorescent quantitative PCR instrument (Applied Biology, USA). The fluorescence signals in each cycle reaction tube were automatically quantified and recorded and the curve was drawn. At the end of the reaction, the results were analyzed by SDS software, and the quantitative values were automatically calculated.

Reaction system: the primers used are shown in **Table 9** (20 μL/tube).

Reaction conditions: ① 95°C for 5 minutes; ② 40 cycles of 95°C for 30 seconds, 55°C for 30 seconds, and 72°C for 35 seconds; and ③ 72°C for 8 minutes.

TABLE 9︱Primer sequences.

| Gene | Accession No. | Primer sequence | | Primer positioning (Start) | Primer length (bp) | Amplification product length (bp) |
| --- | --- | --- | --- | --- | --- | --- |
| GAPDH | NM_008084 | Upstream | 5’ACCCAGAAGACTGTGGATGG3’ | 594 | 20 | 171 |
|  |  | Downstream | 5’CACATTGGGGGTAGGAACAC3’ | 764 | 20 |  |
| PGC-1α | NM_031347 | Upstream | 5’GTGGATGAAGACGGATTGCC3’ | 336 | 20 | 219 |
|  |  | Downstream | 5’GGTGTGGTTTGCATGGTTCT3’ | 554 | 20 |  |
| NRF1 | NM_001100708 | Upstream | 5’AGCTCTTTGAGACCCTGCTT3’ | 201 | 20 | 234 |
|  |  | Downstream | 5’TGCCGTGGAGTTGAGTATGT3’ | 434 | 20 |  |
| mtTFA | NM_031326 | Upstream | 5’ATCATGACGAGTTCTGCCGT3’ | 1022 | 20 | 151 |
|  |  | Downstream | 5’AGAACTTCACAAACCCGCAC3’ | 1172 | 20 |  |
| PPARα | NM_013196 | Upstream | 5’CGGAATTTGCCAAGGCTAT3’ | 1240 | 19 | 132 |
|  |  | Downstream | 5’TCAGCATCCCGTCTTTGTT3’ | 1371 | 19 |  |

**2.4 Calculation Method**

All of the measured data are expressed as mean ± standard deviation ($\bar{x}$± *s*). Quantitative RT-PCR was based on amplification fold, and the results were calculated by the 2^−ΔΔCt^ method as follows: 2^−ΔΔCt^ = (Ct_target gene_ − Ct_housekeeping gene_) experimental group − (Ct_target gene_ − Ct_housekeeping gene_) control group.

**3 The Protein Expression Levels of PGC-1α, PPARα, NRF1, and mtTFA Analyzed by Western blot**

**3.1 Protein Extraction**

RIPA protein extraction reagent was precooled and protease inhibitor cocktail (Roche) was added. Rat myocardial tissue was homogenized in RIPA lysis solution (10% w/v). After incubation on ice for 20 minutes, samples were centrifuged at 13,000 rpm (4°C) for 20 minutes. The supernatant was aliquoted and stored at −80°C for further analysis.

**3.2 Protein Quantitation by BCA**

Following the instructions of the BCA protein quantification kit, the protein concentration was determined on the basis of a standard curve (**Figure 24**).

FIGURE 24︱Standard curve of BCA protein concentration.

The protein concentration was adjusted to a final value of 3.5 μg/μL by dilution in RIPA buffer. Before Western blot analysis, 5× protein sample buffer was added and samples were heated for 5 minutes at 95°C.

**3.3 Western Blot Assay**

① According to the molecular weight of the target protein, 10% separation gels and 5% stacking gels were used.

② For electrophoresis, 35 μg protein was loaded per lane.

③ Electrophoresis conditions: for stacking, a voltage of 90 V was applied for about 20 minutes; for separation, a voltage of 120 V was applied. The electrophoresis stop time depended on the migration of the pre-stained protein marker.

④ Proteins were transferred to a 0.45 μm pore size PVDF membrane at 300 mA for 100 minutes. After transfer, the membrane was dyed with staining reagent to confirm protein transfer was successful.

⑤ The membrane was blocked by complete immersion in 5% BSA-TBST and shaking for 1 hour at room temperature.

⑥ The membrane was incubated with primary antibody in 5% BSA-TBST overnight at 4°C in a horizontal shaker.

⑦ The membrane was washed three times with TBST for 10 min.

⑧ The membrane was incubated with HRP-conjugated goat anti-rabbit IgG (H+L) or HRP-conjugated goat anti-mouse IgG (H+L) (both 1:10,000) in 5% BSA-TBST at room temperature for 40 minutes and subsequently washed three times with TBST for 10 min.

⑨ ECL was titrated onto the surface of the membrane and after 3–5 minutes, the film was exposed for 10 seconds to 5 minutes (exposure time was adjusted on the basis of the light intensity). After development and fixation, the films were imaged.

⑩ The signal intensity of each specific band was analyzed by ImageJ software. The gray value of the target protein was divided by the gray value of the internal reference (GAPDH) to calculate the relative levels of the target protein.

**4 Mitochondrial DNA Detection**

**4.1 DNA Extraction**

DNA was extracted using a rapid DNA extraction kit (TIANcombi DNA Lyse&Det PCR Kit, batch number: N3114, Tiangen Biochemical Technology [Beijing] Co., Ltd.). ① Rat myocardial tissue (2 mg) was collected in a 1.5 mL centrifuge tube, 100 μL buffer B1 was added (ensuring that the buffer completely covered the sample), and the sample was meshed with a grinding pestle. ② Buffer B2 (100 μl) was added, and samples were mixed well and centrifuged at 12,000 rpm for 2 minutes. ③ After centrifugation (High-speed refrigerated centrifuge, HITACHI company), 100 μl of supernatant (template) was carefully pipetted into another 1.5 ml centrifuge tube. ④ The PCR amplification reaction was carried out.

**4.2 PCR Reaction**

SYBR Green PCR Master Mix kit (batch number: 1402443, Applied Biology, USA) was used for PCR on a 7900HT fluorescent quantitative PCR instrument (Applied Biology, USA). The PCR reaction system is shown in **Table 10**.

TABLE 10︱The PCR reaction system.

| Component | Amount |
| --- | --- |
| 2× SyBr Green Mix | 10 μL |
| Forward primer (10 μM) | 1 μL |
| Reverse primer (10 μM) | 1 μL |
| Template DNA | 1 μL |
| RNase-Free ddH_2_O | Add up to 20 μL |

The reagents were added, and samples were mixed well and centrifuged immediately to collect all the reagents at the bottom of the tube.

Reaction conditions: were as follows: ① 94°C for 3 minutes; ② 35 cycles of 94°C for 30 seconds, 55°C for 30 seconds, and 72°C for 60 seconds; and ③ 72°C for 5 minutes. The primers used are shown in **Table 11**.

TABLE 11︱Primer sequences.

| Gene | Primer sequence | |
| --- | --- | --- |
| CytB | Upstream | 5’GCAGCTTAACATTCCGCCCAATCA3’ |
|  | Downstream | 5’TACTGGTTGGCCTCCGATTCATGT3’ |
| RPL10A | Upstream | 5’TTGAAGGCAGCAACAAGGTTTA3’ |
|  | Downstream | 5’GAAGATGACCGATGACGAGCTA3’ |

**4.3 Calculation method**

Relative RNA levels were calculated with the following formula: 2^−ΔΔCt^ = (Ct_target gene_ − Ct_housekeeping gene_) experimental group − (Ct_target gene_ − Ct_housekeeping gene_) control group.
